# Palladium [^*t*‑Bu^(PCP)PdOH] Pincer Complex as a Catalyst in the Michael Reaction

**DOI:** 10.1021/acsomega.5c09674

**Published:** 2026-01-28

**Authors:** Matic Urlep, Matic Lozinšek, Janez Cerkovnik

**Affiliations:** a Faculty of Chemistry and Chemical Technology, University of Ljubljana, Večna pot 113, Ljubljana 1000, Slovenia; b Jožef Stefan Institute, Jamova cesta 39, Ljubljana 1000, Slovenia

## Abstract

The Michael reaction is a versatile carbon–carbon bond-forming strategy that is efficiently catalyzed by transition metal complexes. We report that the palladium [^*t‑*Bu^(PCP)­PdOH] pincer complex effectively activates substrates and forms stable complexes with ketones. The reactive intermediate [^*t‑*Bu^(PCP)­Pd­(acac)] complex, which is formed *in situ* from acetylacetone and the [^*t‑*Bu^(PCP)­PdOH] complex, was successfully isolated and structurally characterized by single-crystal X-ray diffraction. This complex serves as an active catalyst in the reaction between acetylacetone and various *trans*-nitrostyrenes, providing the corresponding Michael adducts in high isolated yields (87–98%). The reaction proceeds under mild conditions with a low catalyst loading, short reaction times, and often minimal or no solvent. These results show that PCP-palladium pincer complexes can serve as efficient, practical catalysts for C–C bond-forming condensation reactions.

## Graphical abstract

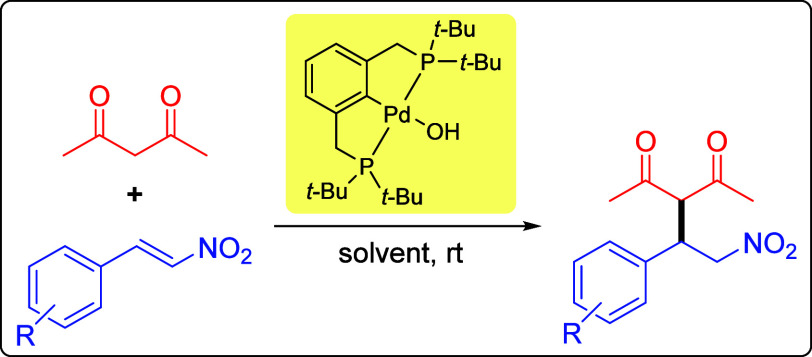

## Introduction

The conjugate addition of carbon nucleophiles to electron-deficient alkenes is one of the most powerful and versatile strategies for the formation of carbon–carbon bonds.^1^ This transformation is widely used for the synthesis of natural products and pharmaceutically important compounds due to its high efficiency and broad compatibility with functional groups.^1^ Since the pioneering studies on asymmetric Michael reactions catalyzed by metal complexes,^2^ considerable progress has been made in broadening the spectrum of Michael donors and acceptors. Significant efforts have been made to develop transition metal catalysts for stereodivergent syntheses that provide access to highly functionalized chiral building blocks.^3–8^

A well-established model reaction for the evaluation of new catalysts in Michael addition chemistry is the conjugate addition of 2,4-pentanedione (acetylacetone) to *trans*-β-nitrostyrenes. This reaction has been extensively studied, and numerous asymmetric variants have been developed using small-molecule organocatalysts, in particular bifunctional thiourea and squaramide derivatives (Figure 1).^9–24^ These catalysts achieved a high degree of stereoselectivity and operational simplicity. Despite these advances, transition metal-based pincer complexes, which are structurally well-defined and highly tunable, have not been explored for this transformation. Pincer ligands offer exceptional stability and modularity, making them attractive platforms for catalytic development. Palladium pincer complexes have been widely studied in various bond-forming reactions, including cross-coupling and conjugate addition.^25–37^ There are also reports of palladium pincer complexes catalyzing Michael reactions;^38^ however, their active role in the catalytic mechanism has not yet been described.

**Figure 1:**
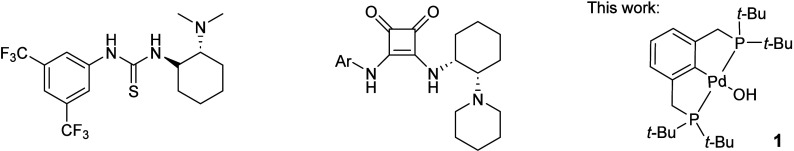
Bifunctional thiourea^10–15,20^ and squareamide^14–16,20^ organocatalysts and the palladium [^*t*‑Bu^(PCP)­PdOH] pincer complex (**1**) used in our study.

Herein, we report the catalytic performance of the palladium [^*t‑*Bu^(PCP)­PdOH] pincer complex (**1**) in the racemic Michael addition of acetylacetone to *trans*-nitrostyrenes. The catalyst offers the possibility of combining strong σ-donor properties with the potential for substrate activation through proton abstraction and metal coordination and promotes efficient C–C bond formation under mild conditions with excellent yields over a wide range of substrates.

## Results and Discussion

### Investigation of the Catalytic Properties of [^*t*‑Bu^(PCP)­PdOH] Complex (**1**)

Dissolution of the palladium [^*t*‑Bu^(PCP)­PdOH] pincer complex (**1**) in acetone-*d*_6_ led to rapid H/D exchange between the residual H_2_O and the solvent, as shown by ^1^H NMR spectroscopy (Figure 2). After the addition of H_2_O to the solution, the resonance of added H_2_O disappears, and a new proton multiplet appears in the methyl region of the solvent signal. These changes observed on the NMR time scale under the conditions used indicate that complex **1** behaves like a Brønsted base and can catalyze H/D exchange.

**Figure 2:**
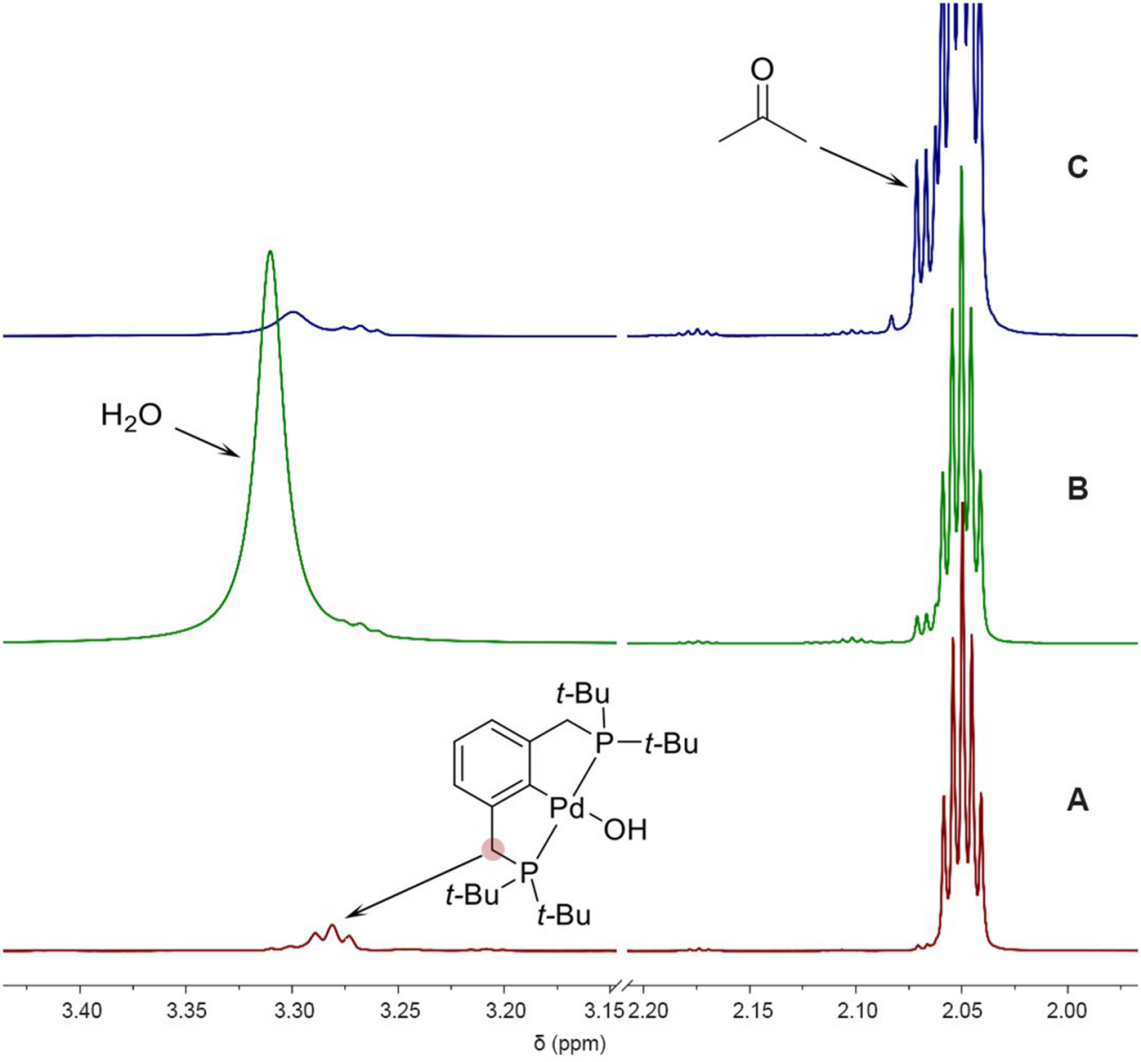
Sections of the ^1^H NMR spectra of complex **1** (6 μM in acetone-*d*_6_): (A) initial solution of complex **1**; (B) after addition of H_2_O (20 μL); (C) after 12 h at room temperature. The spectra show the disappearance of the H_2_O resonance and the appearance of an additional proton multiplet in the methyl region of acetone, consistent with the H/D exchange between H_2_O and acetone-*d*_6_.

Inspired by this unexpected reactivity, we investigated the reaction of complex **1** with other ketone substrates as potential nucleophilic sources. The addition of acetylacetone (acac, **2**) to a solution of complex **1** in acetone-*d*_6_ led to the appearance of a new set of signals in the ^1^H and ^31^P NMR spectra, which we tentatively assigned to the *in situ* formed complex adduct [^*t*‑Bu^(PCP)­Pd­(acac)] (**3**; Figure 3). Subsequently, when *trans*-nitrostyrene (**4a**), a representative Michael acceptor, was added to a solution of the isolated complex **3**, a new set of resonances quickly appeared, which were assigned to the product (**6a**) from the conjugate addition of acetylacetone to *trans*-nitrostyrene (Scheme 1).

**Figure 3:**
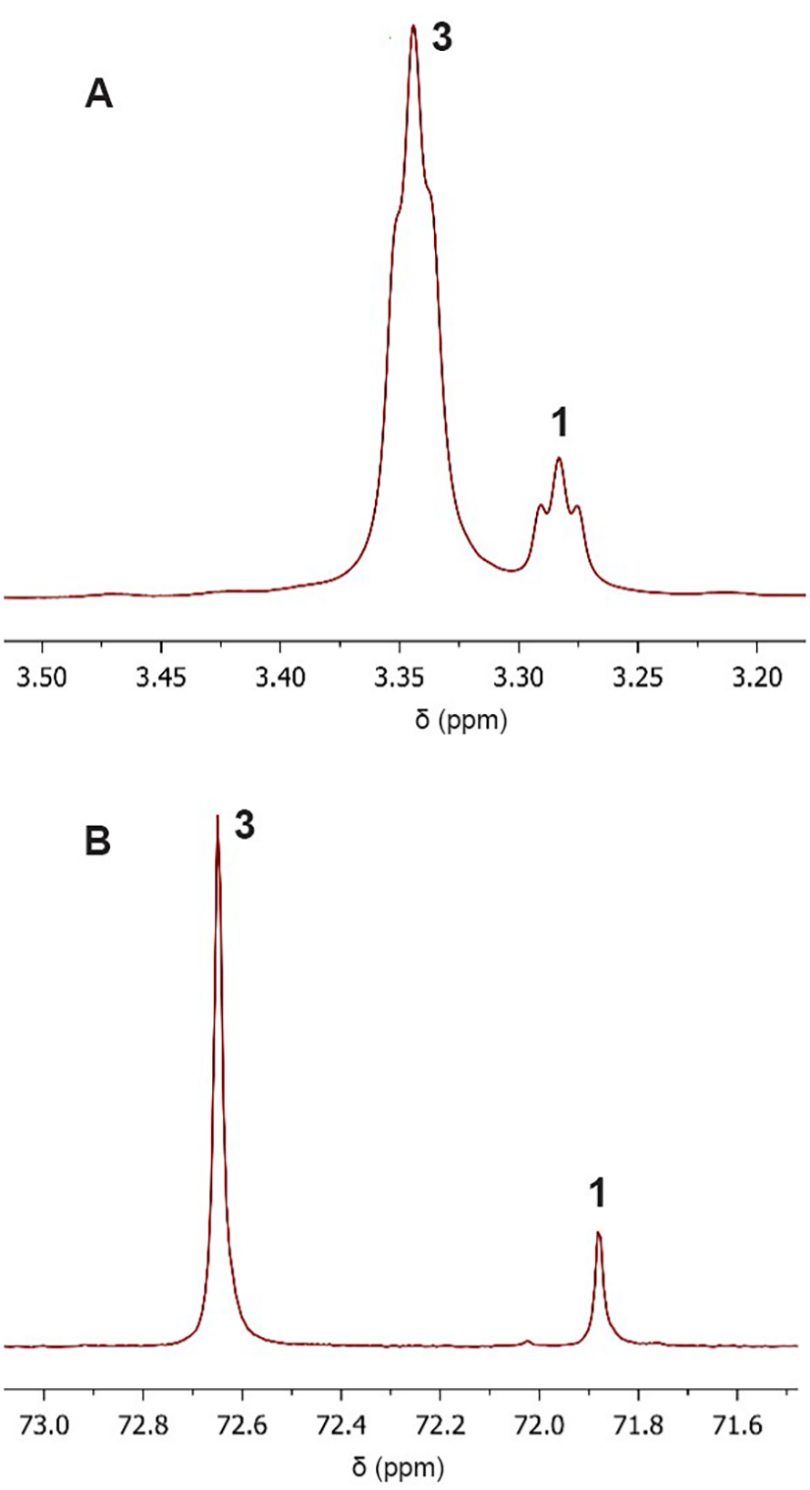
Sections of the ^1^H and ^31^P NMR spectra recorded in acetone-*d*_6_ at room temperature showing the equilibrium between [^*t*‑Bu^(PCP)­PdOH] (**1**) and [^*t*‑Bu^(PCP)­Pd­(acac)] (**3**). (A) ^1^H NMR region highlighting the methylene protons of the [^*t‑*Bu^(PCP)­PdX] series (**1** and **3**). (B) ^31^P NMR region showing the phosphorus resonances of complexes **1** and **3**.

**Scheme 1:**
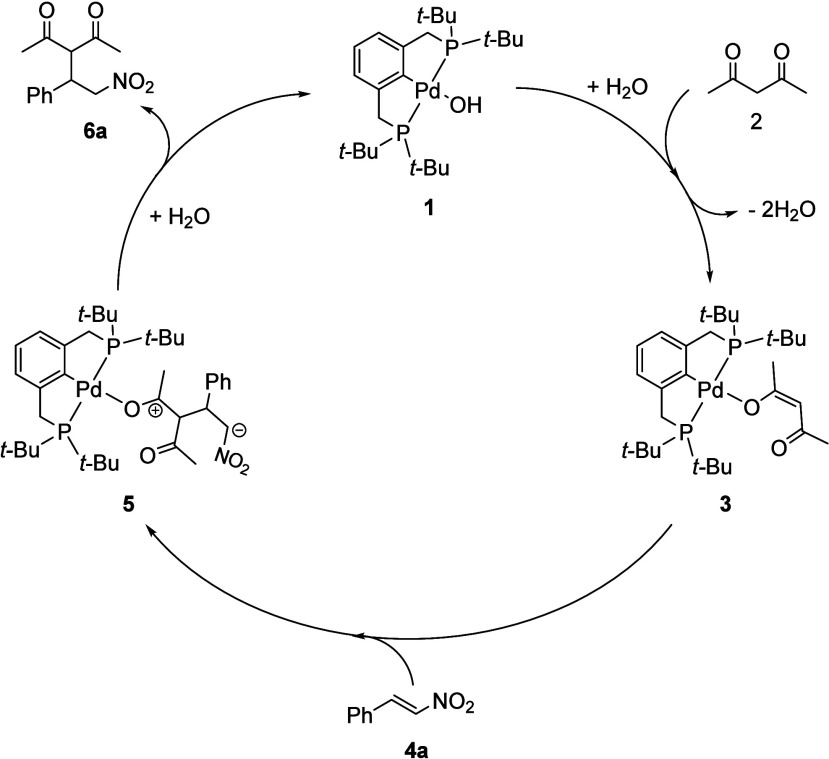
Proposed Catalytic Cycle for the Michael Addition of Acetylacetone (**2**) to *trans*-Nitrostyrene (**4a**) Mediated by [^*t*‑Bu^(PCP)­PdOH] Complex (**1**)­^a^

a Water-assisted formation of the [^*t*‑Bu^(PCP)­Pd­(acac)] complex (**3**) is followed by reaction with **4a** to form the intermediate complex **5**, and water-assisted protonation and product release furnish **6a** and regenerate complex **1**.

Control experiments indicate that H_2_O acts as a cocatalyst in the formation of complex **3** and in catalyst turnover. When acetylacetone (**2**) was added to complex **1** in dry acetone-*d*_6_, the formation of complex **3** could not be observed by ^1^H NMR, indicating that water is required for the formation of active complex **3**. Based on these observations, we propose a water-mediated pathway in which an initial proton transfer involving H_2_O promotes the conversion of the [^*t*‑Bu^(PCP)­PdOH] complex (**1**) and acetylacetone (**2**) to the acac-bound [^*t*‑Bu^(PCP)­Pd­(acac)] complex (**3**) with the concomitant release of H_2_O. Complex **3** then reacts with **4a** to form intermediate complex **5**, which, after protonation and water-assisted product release, gives **6a** and regenerates complex **1** (Scheme 1).

The importance of water for catalysis was further confirmed by comparative ^1^H NMR experiments: In the absence of added water, the reaction of **2** with **4a** proceeds sluggishly in the presence of complex **1** (∼10% conversion to **6a** after 12 h at room temperature, which can be attributed to traces of water in the in commercially available solvent), whereas the addition of 10 equiv of H_2_O accelerates the near-quantitative conversion to **6a** within 12 h under identical conditions (0.5 mol % of complex **1**, room temperature) (Figure 4).

**Figure 4:**
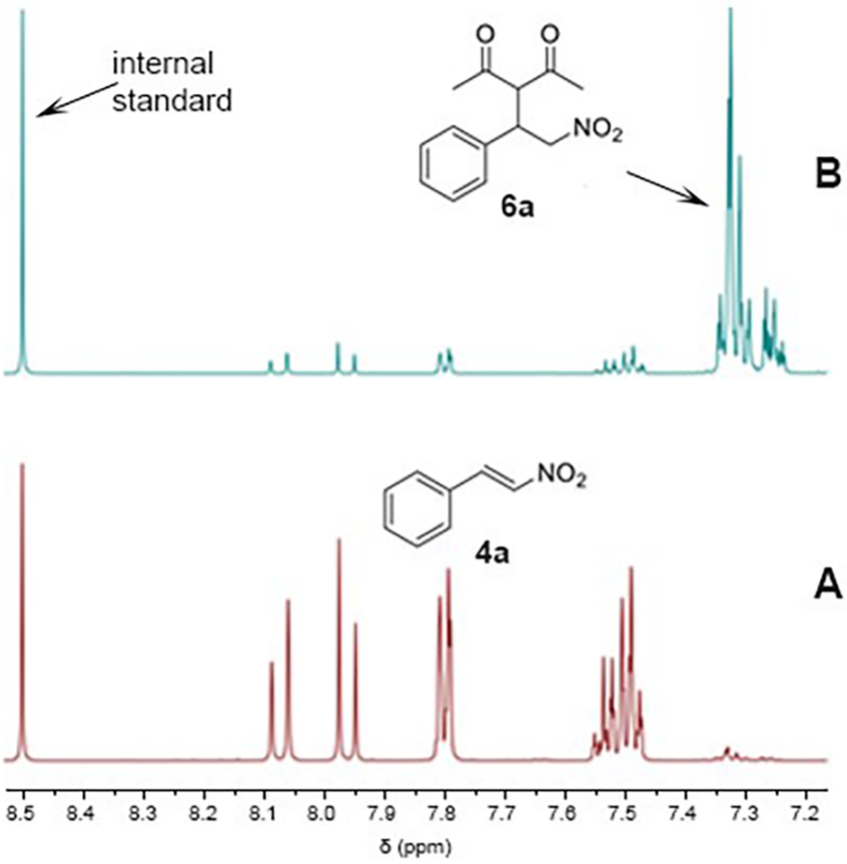
^1^H NMR spectra of mixtures of acetylacetone (**2**) and *trans*-nitrostyrene (**4a**) in acetone-*d*_6_ after 12 h at room temperature: (A) without addition of H_2_O (≈10% conversion to **6a**) and (B) after addition of 10 equiv of H_2_O (almost quantitative conversion to **6a**). Catalyst loading, 0.5 mol %.

Taken together, these data show that the [^*t*‑Bu^(PCP)­PdOH] complex (**1**) mediates rapid H/D exchange between H_2_O and acetone-*d*_6_ and promotes the activation of acetylacetone for Michael addition, with water playing a crucial role in the formation of the active species and in product release.

### Isolation of the [^*t‑*Bu^(PCP)­Pd­(acac)] Complex (**5**)

To test the mechanistic hypothesis, the [^*t*‑Bu^(PCP)­Pd­(acac)] complex (**3**) was synthesized (Scheme 2, see the Supporting Information for experimental details), structurally characterized, and used as a catalyst. Complex **3** was crystallized from wet acetylacetone by the addition of hexane and yielded single crystals of the solvates **3**·0.5acac and **3**·0.5C_6_H_14_. These were analyzed by low-temperature single-crystal X-ray diffraction (Figure 5, see the Supporting Information for details). The solvent molecules acac and hexane are positioned on an inversion center, with acac disordered over two equally occupied positions. The molecular geometries of complex **3** in both crystal structures are nearly identical; here, the structure of **3**·0.5acac is discussed, while details on both structures are provided in the Supporting Information. Palladium adopts a slightly distorted square-planar coordination geometry with the following bond lengths: Pd–C 2.0105(16) Å, Pd–O 2.1050(12) Å, and Pd–P 2.3172(4) and 2.3241(4) Å. The P–Pd–P and C–Pd–O angles deviate from the ideal 180° linearity, measuring 167.210(16) and 168.66(6)°, respectively. The C–Pd–P angles are acute (83.17(5) and 84.06(5)°), whereas the O–Pd–P angles are obtuse (94.94(4) and 97.74(4)). The enol form of the coordinated acetylacetonate ligand adopts an anti configuration, with the following bond parameters: C–O­(Pd) 1.286(2) Å, C=O 1.236(2) Å, C=C 1.374(3) Å, C–C 1.430(3) Å, and C–CH_3_ 1.507(2) and 1.519(3) Å.

**Scheme 2:**
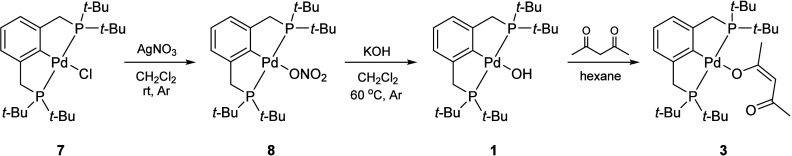
Synthesis of the [^*t‑*Bu^(PCP)­Pd­(acac)] Complex (**3**)

**Figure 5:**
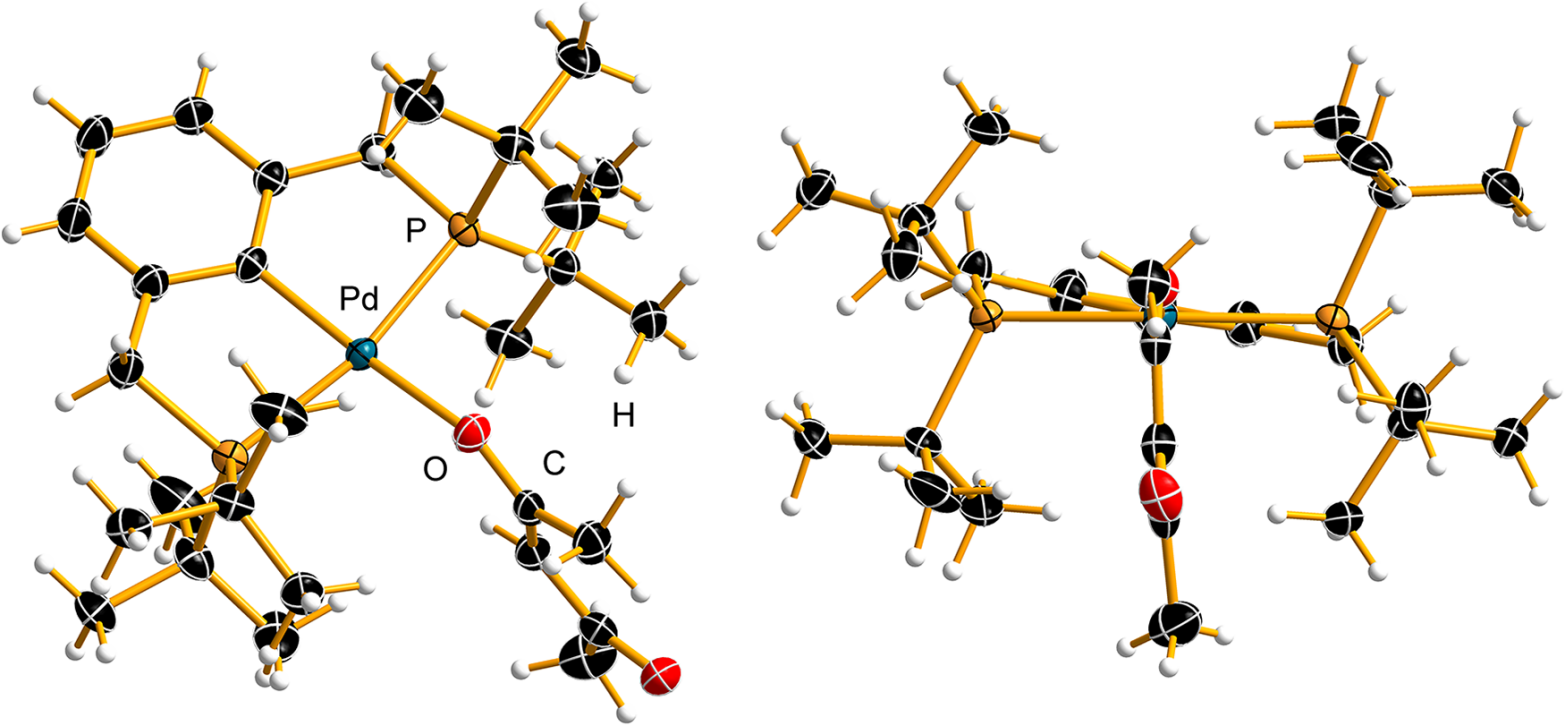
X-ray crystal structure of the [^*t‑*Bu^(PCP)­Pd­(acac)] complex (**3**) from the cocrystal **3**·0.5acac. The thermal ellipsoids are shown with 50% probability, and the hydrogen atoms are shown as spheres of the arbitrary radius.

If the proposed catalytic cycle works, then acetylacetone-bound complex **3** should release [^*t*‑Bu^(PCP)­PdOH] (**1**) as the active catalyst. In agreement with this hypothesis, treatment of isolated complex **3** with *trans*-nitrostyrene (**4a**) led to the formation of Michael product **6a**, which was accompanied by a transient regeneration of complex **1**, as observed by ^1^H NMR. The intermediates involved in this process were detected and characterized by combined ^1^H and ^31^P NMR spectroscopy (Figure 6). X-ray quality crystals of [^*t*‑Bu^(PCP)­Pd­(acac)] complex (**3**) were dissolved in an NMR tube in commercially available acetone-*d*_6_ (which always contains some water) at −10 °C, and equilibrium between complexes **1** and **3** was rapidly established (Figure 6, spectrum A). The addition of 1.0 equiv of **4a** to this mixture led to a new equilibrium within 60 min at −10 °C; the simultaneous appearance of new resonances in the ^1^H and ^31^P spectra is assigned to the most likely intermediate **5** (Figure 6, spectrum B). After addition of an excess of acetylacetone (**2**; 20 equiv), the system preferentially returned to the equilibrium between complexes **1** and **3** and the signals attributed to intermediate **5** decreased markedly as **4a** is almost consumed (Figure 6, spectrum C). Because the species remain in equilibrium under these conditions, resonances attributable to transient intermediate **5** do not disappear completely.

**Figure 6:**
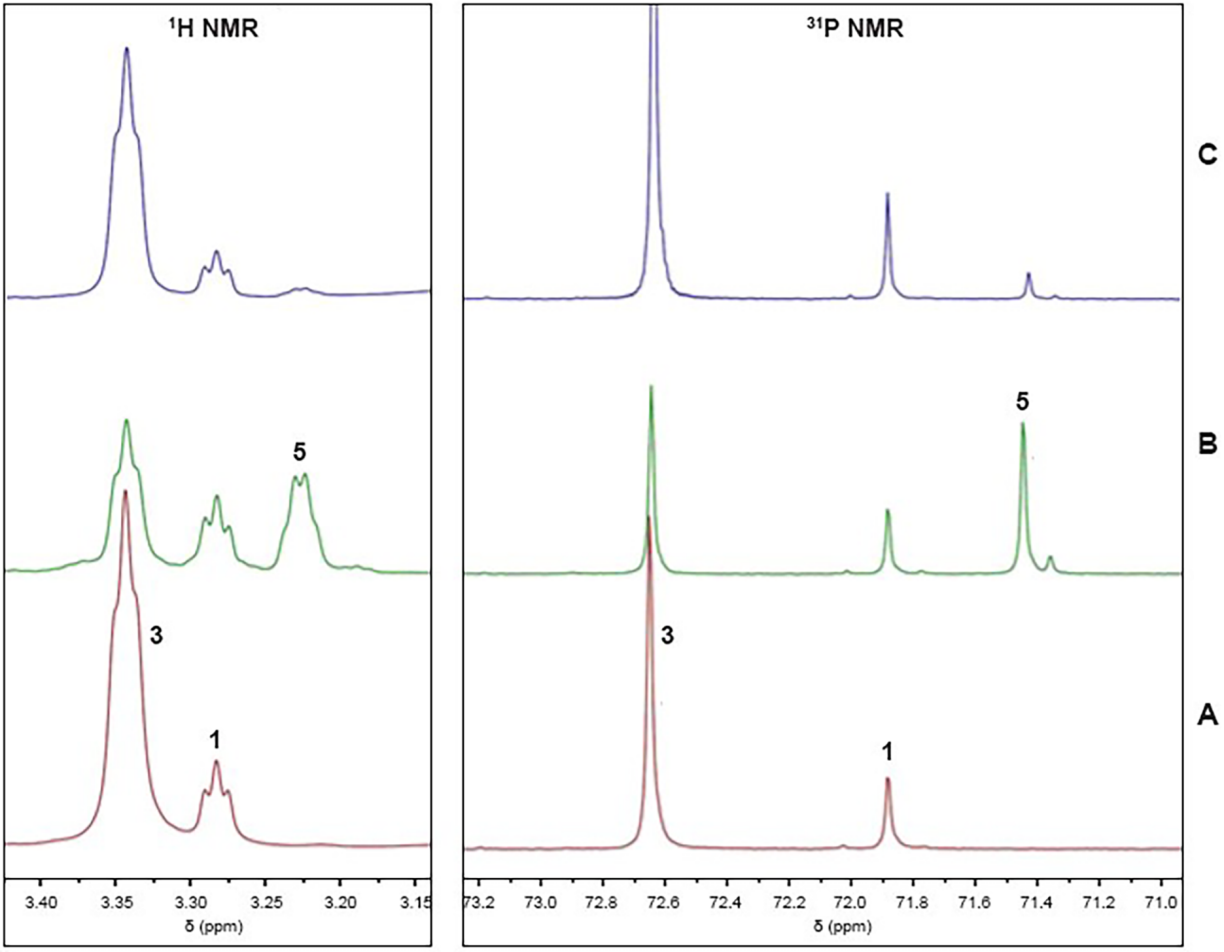
Sections of the ^1^H and ^31^P NMR spectra recorded at −10 °C in commercially available acetone-*d*_6_, which always contains some water, showing the intermediates observed in the catalytic process. The ^1^H NMR resonances shown correspond to the methylene protons of the [^*t‑*Bu^(PCP)­PdX] complexes (**1**, **3**, and **5**), and ^31^P NMR resonances shown correspond to the phosphorus resonances of complexes **1**, **3**, and **5**. (A) Solution of isolated complex **3** in acetone-*d*_6_ showing rapid equilibrium between complexes **1** and **3**; (B) after addition of 1.0 equiv of *trans*-nitrostyrene (**4a**) with new ^1^H and ^31^P signals assigned to intermediate **5**; (C) after addition of 20 equiv of acetylacetone (**2**), showing a restoration of equilibrium between complexes **1** and **3** and a decrease in signals attributable to transient complex **5**.

The propensity of the catalyst to promote H/D exchange was further investigated by treating a mixture of the Michael product **6a** and the isolated [^*t*‑Bu^(PCP)­Pd­(acac)] complex (**3**) in acetone-*d*_6_ with 20 equiv of D_2_O. After 24 h, when equilibrium was reached, the ^1^H NMR spectrum showed the complete disappearance of the acidic proton signal (H_b_) and a clear attenuation of the benzylic proton signal (H_c_) (Figure 7), indicating the incorporation of deuterium at both positions. These observations indicate that exchange at the acidic positions of **6a** is readily possible under the reaction conditions and that the benzylic site is also susceptible to partial H/D exchange, consistent with metal-assisted deprotonation/enolization or proton transfer processes mediated by the palladium complex.

**Figure 7:**
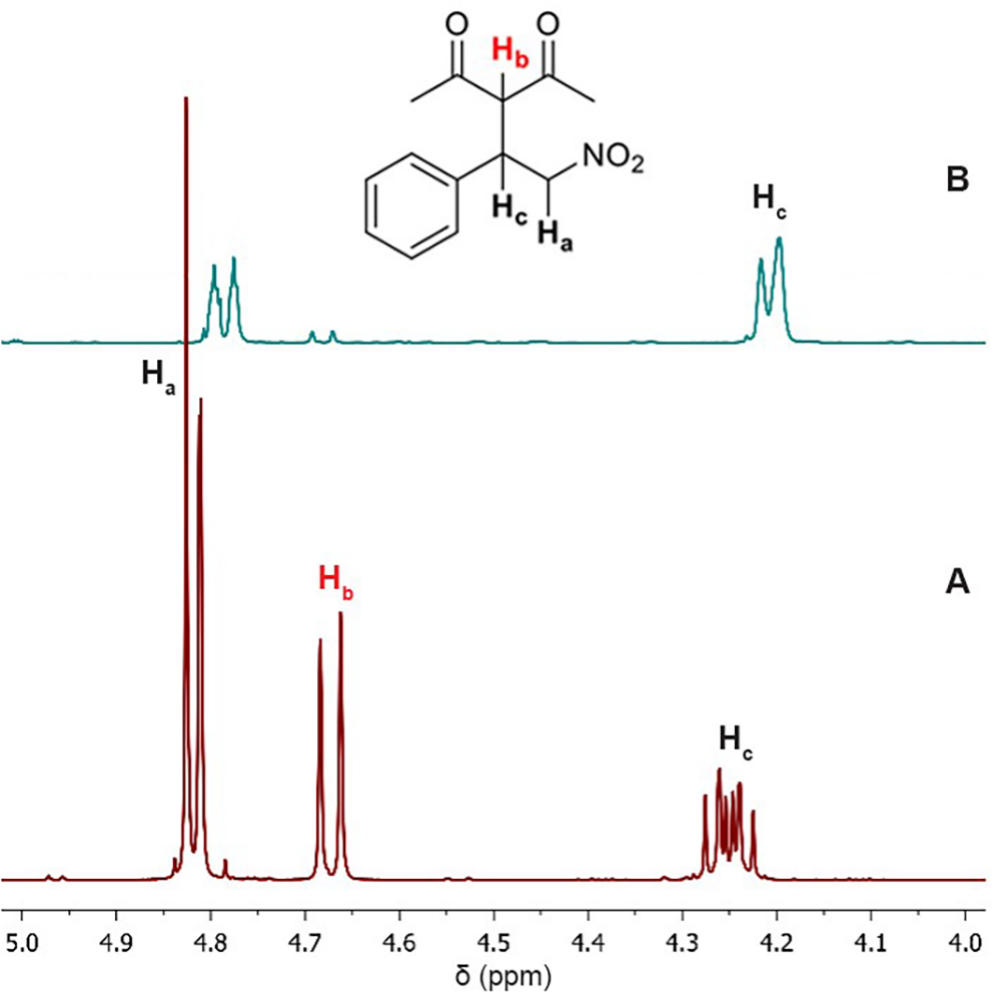
^1^H NMR monitoring of H/D exchange in acetone-*d*_6_: (A) initial mixture of product **6a** and [^*t‑*Bu^(PCP)­Pd­(acac)] complex (**3**) in dry acetone-*d*_6_; (B) after 24 h following the addition of 20 equiv of D_2_O, showing the disappearance of the acidic proton (H_b_) and a marked reduction in the signal of the benzylic proton (H_c_), indicating the incorporation of deuterium.

### Optimization of the Reaction Conditions

More stable acetylacetone-bound [^*t*‑Bu^(PCP)­Pd­(acac)] complex (**3**) was used as a catalyst to optimize the conjugate addition of acetylacetone (**2**) to *trans*-nitrostyrene (**4a**). Solvent screening with 10 mol % catalyst loading showed a pronounced solvent dependence. The reactions in chlorinated solvents (Table 1, entries 1 and 2) proceeded very slowly, which can be attributed to the deactivation of the catalyst by traces of HCl (formation of the chlorine-substituted complex, e.g., **7**). THF also gave poor conversions, while toluene performed better but required prolonged heating to give appreciable conversion (Table 1, entry 4). Acetone proved to be the solvent of choice: with 10 mol % of complex **3**, the reaction achieved a quantitative conversion in less than 1 h (Table 1, entry 6).

**Table 1:**
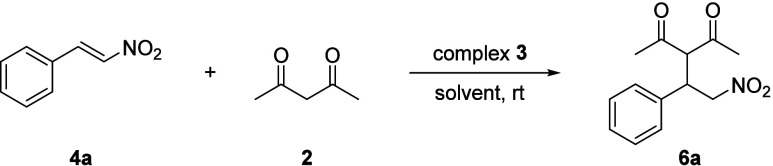
Optimization of Reaction Conditions^a^

entry	solvent	**solvent volume** [mL]	**catalyst** [mol %]	**react. time** [h]	**conversion** [%]
1	DCM	1	10	24	5
2	CHCl_3_	1	10	24	11
3	toluene	1	10	24	20
4	toluene^b^	1	10	24	80
5	THF	1	10	24	7
6	acetone	1	10	<1	100
7	acetone	1	5	8	100
8	acetone	1	1.5	12	100
9	acetone	1	0.5	24	100
10	acetone	0.5	1.5	8	100
11	acetone	0.05	0.5	10	100
12	acetone	0.05	1.5	1	100
13		solvent-free	1.5	1	100
14^c^	acetone	1	1.5	12	95
15^d^	acetone	0.05	1.5	3	97
16^e^	acetone	0.05	1.5	2	98
17^f^	acetone	1	10	24	80

a Reactions were carried out using 0.25 mmol of *trans*-nitrostyrene (**4a**) and 0.5 mmol of acetylacetone (**2**) at room temperature. Under these conditions, pincer complexes **1** and **3** remained stable, with no evidence of palladium black formation or Pd leaching.

b *T* = 80 °C.

c Benzoylacetone was used instead of **2** in this reaction.

d Ethyl acetoacetate replaced **2**.

e The reaction was carried out with nitromethane instead of **2**.

f The reaction was carried out with [^*t‑*Bu^(PCP)­PtOH], a platinum analogue of complex **1**, as the catalyst.

The catalyst loading could be significantly reduced without a loss of activity. Near-quantitative conversion was routinely achieved with a 1.5 mol % catalyst and 0.05 mL of acetone under conditions optimized for scope evaluation (0.25 mmol of **4a** and 0.50 mmol of **2**; Table 1). The reactions can also be carried out solvent-free by using 2.0 equiv of acetylacetone to dissolve **4a**. However, this approach led to poor conversions for several substituted *trans-*nitrostyrenes, as the solubility of these substrates was significantly reduced. Quantitative conversions were obtained for a range of *trans*-nitrostyrenes with different functional groups using 1.5 mol % of the catalyst in the presence of 0.05 mL of acetone, although substrates with an increased number of functional groups on the aryl ring reacted more slowly. The reduced rate with bulky *trans*-nitrostyrenes is attributed to high steric demand of the *tert*-butyl groups on the PCP ligand of the catalyst, which likely hinders the approach of the nucleophile or the binding of the substrate to the metal center.

We also investigated benzoylacetone, ethyl acetoacetate, and nitromethane as pronucleophiles in the conjugate addition to model *trans*-nitrostyrene under conditions optimized for scope evaluation. Benzoylacetone reacted efficiently, although with slightly longer reaction times (Table 1, entry 14), while ethyl acetoacetate and nitromethane exhibited effectiveness comparable to that of acetylacetone (**2**), as detailed in Table 1 (entries 15 and 16). Finally, the platinum analogue of complex **1** was briefly evaluated (Table 1, entry 17); however, it displayed much lower catalytic activity and was not pursued further.

The palladium pincer complex **3** efficiently catalyzes the conjugate addition of acetylacetone (**2**) to a wide range of *trans*-nitrostyrenes (**4**) and provides the corresponding Michael adducts in high isolated yields (87–98%; Table 2). The catalyst also promotes the addition to nitropent-2-ene and provides the product in 85% isolated yield (Table 2, entry 20). Low catalyst loadings, short reaction times, and the ability to perform reactions with minimal solvent or under solvent-free conditions highlight the practical advantages of these pincer complexes for the scalable formation of carbon–carbon bonds (see Table 2 and the Supporting Information for the experimental details and characterization).

**Table 2:**
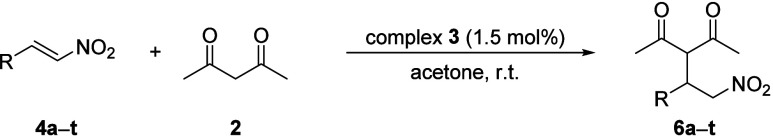
Substrate Scope^a^ ^,^ ^b^

entry	**nitrostyrene,** R	***t*** [h]	**product,** yield [%]^c^
1	**4a**, Ph	<1	**6a**, 94
2	**4b**, 4-Cl-C_6_H_4_	2	**6b,** 99
3	**4c**, 4-Br-C_6_H_4_	2	**6c**, 96
4	**4d**, 4-OMe-C_6_H_4_	4	**6d**, 96
5	**4e**, 4-Me-C_6_H_4_	1	**6e**, 98
6	**4f**, 4-CN-C_6_H_4_	1	**6f**, 95
7	**4g**, 2-F-C_6_H_4_	1	**6g**, 98
8	**4h**, 2-Cl-C_6_H_4_	2	**6h**, 99
9	**4i**, 2-Br-C_6_H_4_	2	**6i**, 96
10	**4j**, 2-OMe-C_6_H_4_	3	**6j**, 97
11	**4k**, 2-NO_2_-C_6_H_4_	1	**6k**, 98
12	**4l**, 3-Cl-C_6_H_4_	8	**6l**, 99
13	**4m**, 3-Br-C_6_H_4_	8	**6m**, 97
14	**4n**, 3-OMe-C_6_H_4_	10	**6n**, 87
15	**4o**, 2,6-Cl_2_-C_6_H_4_	4	**6o**, 94
16	**4p**, 2,4-Cl_2_-C_6_H_4_	5	**6p**, 95
17	**4q**, 2,5-MeO_2_-C_6_H_4_	5	**6q**, 96
18	**4r**, 3,4,5-MeO_3_-C_6_H_4_	6	**6r**, 92
19	**4s**, 2-furyl	1	**6s**, 98
20	**4t**, Pr	5	**6t**, 85

a Reactions were carried out at the scale of 0.25 mmol of *trans*-nitrostyrene (**4**), with acetylacetone (**2**, 0.50 mmol), complex **3** (1.5 mol % relative to **4**), and acetone (50 μL) at room temperature.

b Using methyl acrylate as the Michael acceptor under the same conditions resulted in a 96% yield.

c Isolated yields refer to the products purified by flash chromatography (hexane/EtOAc = 2:1).

The reaction of unsubstituted *trans*-nitrostyrene (**4a**) with acetylacetone was much faster than the reactions of substituted *trans*-nitrostyrenes. The introduction of electron-withdrawing substituents (e.g., −CN, −Br, and −Cl) did not accelerate the reaction but even led to slower reaction rates. A plausible explanation is the steric strain caused by the *tert*-butyl groups on the PCP ligand: Bulky *tert*-butyl substituents on the phosphorus probably hinder the approach of substituted *trans*-nitrostyrene to the metal center, thereby reducing the rate of substrate coordination and conversion.

Attempts to determine a kinetic isotope effect were inconclusive, as the H/D exchange between complex **1**, acetylacetone, and added D_2_O is exceptionally fast under the reaction conditions. The addition of 20 equiv of D_2_O to a solution of complex **1** and acetylacetone in pure, dry acetone-*d*_6_ produced an instantaneous H_2_O signal in the ^1^H NMR spectrum, consistent with rapid H/D exchange and involvement of exchanged protons in the catalytic process.

The reaction of *trans*-nitrostyrene (**4a**) was also carried out at gram scale under the conditions in Table 1 (entry 11), and the recrystallized product **6a** was isolated in 87% yield.

## Conclusions

We demonstrate that the palladium pincer [^*t*‑Bu^(PCP)­PdOH] complex (**1**) efficiently activates acetylacetone (acac) for Michael addition to *trans*-nitrostyrenes. The intermediate acac-bound [^*t*‑Bu^(PCP)­Pd­(acac)] complex (**3**), formed *in situ* from complex **1** and acetylacetone, was isolated and unambiguously characterized by single-crystal X-ray diffraction. Key reaction intermediates and catalyst roles were identified and corroborated by ^1^H and ^31^P NMR spectroscopy. Rapid H/D exchange experiments implicate proton transfer and the presence of water as important contributors in the catalytic process. Using isolated complex **3** as the catalyst, the conjugate addition of acetylacetone to a variety of *trans*-nitrostyrenes furnished the corresponding Michael adducts in high isolated yields (87–98%). The protocol tolerates low catalyst loadings, short reaction times, and minimal or solvent-free conditions and is amenable to gram-scale synthesis, indicating that PCP-palladium pincer complexes can serve as practical and robust catalysts for C–C bond-forming condensations.

## Methods

Reagents and solvents were obtained from Merck, Fluorochem, or ABCR Chemicals and used as received. Column chromatography was performed on Fluka Silica Gel 60 (220–240 mesh). Thin- layer chromatography (TLC) was performed on UV_254_ plates and visualized under UV light (254 nm). ^1^H, ^13^C, and ^31^P NMR spectra were recorded in CDCl_3_ or acetone-*d*_6_ using a Bruker Avance III 500 MHz spectrometer (^1^H, 500 MHz; ^13^C, 126 MHz; ^31^P, 202 MHz). Chemical shifts (δ) are given in ppm relative to tetramethylsilane (TMS) for ^1^H and ^13^C and to H_3_PO_4_ (85%) for ^31^P; coupling constants (*J*) are given in Hertz. Abbreviations used: s, singlet; d, doublet; t, triplet; q, quartet; m, multiplet; vt, virtual triplet.

### Synthesis of Palladium [^*t‑*Bu^(PCP)­PdCl] Complex (**7**)

Complex **7** was prepared following the reported procedure, with modifications.^39^ All manipulations were conducted under an inert atmosphere unless otherwise stated. In a dry 100 mL Schlenk flask under argon, 1,3-bis­(di-*tert*-butylphosphinomethyl)­benzene (608 mg, 1.50 mmol) and bis­(benzonitrile)­palladium­(II) chloride (575 mg, 1.50 mmol) were suspended in degassed 2-methoxyethanol (25 mL). The mixture was refluxed under argon for 1 h and then, while still hot, filtered through a Celite pad, washed with CHCl_3_ (3 × 20 mL), and concentrated under reduced pressure. The crude product was purified by flash column chromatography (dichloromethane as an eluent) to give complex **7** as white needle-like crystals in 84% yield (670 mg).

^1^H NMR (500 MHz, CDCl_3_) δ 7.00–6.86 (m, 3H), 3.23 (vt, *J* = 4.0 Hz, 4H), 1.42 (vt, *J* = 6.7 Hz, 36H). ^13^C­{^1^H} NMR (126 MHz, CDCl_3_): δ 140.9, 130.2, 128.5, 126.0, 29.5, 24.1, 18.2. ^31^P NMR (202 MHz, CDCl_3_) δ 72.0. HRMS (ESI-TOF^+^): *m*/*z* calcd for C_24_H_44_ClP_2_Pd^+^ [(M + H)^+^], 535.1638; found, 535.1641.

### Synthesis of Palladium [^*t‑*Bu^(PCP)­PdONO_2_] Complex (**8**)

Complex **8** was prepared by following the reported procedure with modifications.^40^ Complex **7** (100 mg, 0.19 mmol) was dissolved in dry dichloromethane (5 mL) in a dry 25 mL Schlenk flask. The solution was degassed, AgNO_3_ (70 mg, 0.42 mmol, 2.2 equiv) was added, and the mixture was stirred for 18 h at room temperature in the dark. The reaction mixture was filtered through a Celite pad and washed with diethyl ether (3 × 15 mL). The combined organic phases were washed with water (3 × 20 mL), dried over anhydrous Na_2_SO_4_, and concentrated under reduced pressure to give complex **8** as a pale yellow crystalline solid in 98% yield (103 mg).

^1^H NMR (500 MHz, acetone-*d*_6_) δ 7.06–6.79 (m, 3H), 3.35 (vt, *J* = 4.2 Hz, 4H), 1.38 (vt, *J* = 6.9, Hz, 36H). ^13^C­{^1^H} NMR (126 MHz, acetone-*d*_6_): δ 140.9, 130.2, 128.5, 126.0, 30.3, 22.0, 17.1. ^31^P NMR (202 MHz, acetone-*d*_6_) δ 75.0. HRMS (ESI-TOF^+^): *m*/*z* calcd for C_24_H_44_NO_3_P_2_Pd^+^ [(M + H)^+^], 562.1828; found, 562.1830.

### Synthesis of Palladium [^*t‑*Bu^(PCP)­PdOH] Complex (**1**)

Complex **1** was prepared following the reported procedure, with modifications.^40^ Under an argon atmosphere, complex **8** (280 mg, 0.50 mmol) and KOH (138 mg, 2.50 mmol, 5.0 equiv) were added to a dry 25 mL Schlenk flask. The solids were suspended in freshly distilled THF (30 mL), and the mixture was stirred at 60 °C for 20 h. The solvent was removed under reduced pressure, and the residue was extracted with degassed diethyl ether (3 × 25 mL). The concentration of the combined extracts gave complex **1** as a white solid in 89% yield (230 mg).

^1^H NMR (500 MHz, acetone-*d*_6_) δ 6.97–6.72 (m, 3H), 3.28 (vt, *J* = 4.0 Hz, 4H), 1.41 (vt, *J* = 6.8 Hz, 36H). ^13^C­{^1^H} NMR (126 MHz, acetone-*d*_6_): δ 140.9, 130.2, 128.5, 126.0, 30.3, 22.2, 17.3. ^31^P NMR (121 MHz, acetone-*d*_6_) δ 70.4. HRMS (ESI-TOF^+^): *m*/*z* calcd for C_24_H_45_OP_2_Pd^+^ [(M + H)^+^], 517.1979; found, 517.1976.

### Synthesis of Palladium [^*t‑*Bu^(PCP)­Pd­(acac)] Complex (**3**)

Complex **1** (129 mg, 0.25 mmol) was transferred under argon to a 5 mL round-bottomed flask and dissolved in 1 mL of acetylacetone with heating. Then, 3 mL of hexane was layered over the cooled solution to form two layers. The reaction mixture was allowed to stand overnight at room temperature and then transferred to a refrigerator at −30 °C for 1 day. White crystals of complex **3** were obtained in 97% yield (145 mg).

^1^H NMR (500 MHz, acetone-*d*_6_) δ 7.02–6.82 (m, 3H), 5.28 (s, 1H), 3.35 (vt, *J* = 4.1 Hz, 3H), 2.20 (s, 3H), 1.78 (s, 3H), 1.38 (vt, *J* = 6.7 Hz, 36H). ^13^C­{^1^H} NMR (126 MHz, acetone-*d*_6_): δ 191.4, 190.1, 140.9, 130.2, 128.5, 126.0, 100.3, 30.3, 25.2, 23.1, 17.2. ^31^P NMR (202 MHz, acetone-*d*_6_) δ 73.0. HRMS (ESI-TOF^+^): *m*/*z* calcd for C_29_H_51_O_2_P_2_Pd^+^ [(M + H)^+^], 599.2397; found, 599.2399.

## Supplementary Material



## Notes

Additional experimental details, materials, methods, and details of X-ray crystallography, including crystal packing, selected bond distances and angles, and hydrogen-bonding parameters (PDF)

## Notes

CCDC 2432904 and 2432905
