# Supplementary material for: Palladium [ t‑Bu(PCP)PdOH] Pincer Complex as a Catalyst in the Michael Reaction
Source: ACS Omega. 2026 Jan 28;11(5):7833–40. doi: 10.1021/acsomega.5c09674 (PMC12902846; doi:10.1021/acsomega.5c09674)
Supplement: Supplementary file 1 [file ao5c09674_si_001.pdf]

SUPPORTING INFORMATION

The Palladium [$^{t\text{-Bu}}$(PCP)PdOH] Pincer Complex as a Catalyst in the Michael Reaction

Matic Urlep,[a] Matic Lozinšek,[b] and Janez Cerkovnik[a,*]

[a] Faculty of Chemistry and Chemical Technology, University of Ljubljana, Večna pot 113, 1000 Ljubljana, Slovenia

[b] Jožef Stefan Institute, Jamova cesta 39, 1000 Ljubljana, Slovenia

*Email: janez.cerkovnik@fkkt.uni-lj.si

Table of Contents

GENERAL INFORMATION S2

EXPERIMENTAL PART S3

General Procedure for Synthesis of the Products of the Michael Reaction (**6**)........ S3

CRYSTALLOGRAPHIC PART S12

SUPPORTING REFERENCES S20

GENERAL INFORMATION

All reactions were carried out under ambient conditions, if not stated otherwise. Starting materials were used as obtained from commercial sources without further purification (Merck, Fluorochem or ABCR Chemicals). Solvents were used as received and were of technical grade, unless specified otherwise. Column chromatography was performed using Fluka Silica gel 60 (220–240 mesh). Thin-layer chromatography was performed with UV_{254} plates and revealed under UV light. Room temperature refers to 23–25 °C.

NMR spectra were recorded with a Bruker Avance III 500 MHz NMR instrument operating at 500 MHz (^{1}H), 126 MHz (^{13}C) and 202 MHz (^{31}P) in acetone-d_6 and $CDCl_3$ at 296 K. ^{1}H chemical shifts and coupling constants were referenced to the residual signal of $CHCl_3$ (at δ 7.26 ppm) and acetone (at δ 2.05 ppm). Carbon chemical shifts are given against the central line of the solvent signal of $CDCl_3$ (at 77.0 ppm) and ^{31}P chemical shifts are referenced to H_3PO_4 (85%) ($\delta(^{31}P) = 0$ ppm). Coupling constants (*J*) are given in Hz. Multiplicities are indicated as follows: s (singlet), d (doublet), dd (doublet of doublets), ddd (doublet of doublet of doublets), t (triplet), q (quartet), m (multiplet).

EXPERIMENTAL PART

General Procedure for the Synthesis of Products in the Michael Reaction (**6**)

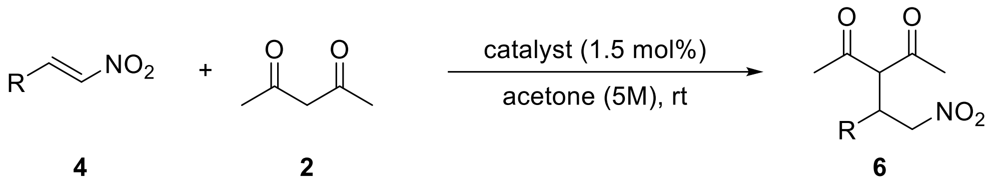

0.22 mmol nitrostyrene (**4**), 2 mg (1.5 mol%) [$^{t\text{-Bu}}$(PCP)Pd(acac)] (**3**), and 46 μL (2 equiv.) acetylacetone (**2**) were weighed into a 2 mL scintillation vial, and then 44 μL acetone was added. The vial was sealed and the reactants were stirred until the reaction was complete. The solvent was then removed under reduced pressure and the product was recrystallized from dichloromethane with hexane overlay, unless otherwise stated.

3-(2-nitro-1-phenylethyl)pentane-2,4-dione (**6a**)[1]

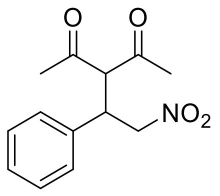

White crystalline solid, 94 % yield.

^{1}H NMR (500 MHz, CDCl$_3$): δ 7.37–7.25 (m, 3H), 7.22–7.16 (m, 2H), 4.69–4.59 (m, 2H), 4.38 (d, *J* = 10.7 Hz, 1H), 4.25 (ddd, *J* = 10.7, 7.7, 5.2 Hz, 1H), 2.28 (s, 3H), 1.94 (s, 3H).

^{13}C{^{1}H} NMR (126 MHz, CDCl$_3$): δ 201.8, 201.0, 136.0, 129.3, 128.5, 128.0, 78.2, 70.6, 42.8, 30.5, 29.7.

3-(1-(4-chlorophenyl)-2-nitroethyl)pentane-2,4-dione (**6b**)[2]

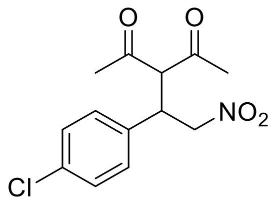

White crystalline solid, 99 % yield.

^{1}H NMR (500 MHz, CDCl$_3$): δ 7.35–7.28 (m, 2H), 7.18–7.10 (m, 2H), 4.65–4.56 (m, 2H), 4.33 (d, *J* = 10.7 Hz, 1H), 4.23 (ddd, *J* = 10.7, 7.0, 5.6 Hz, 1H), 2.29 (s, 3H), 1.98 (s, 3H).

^{13}C{^{1}H} NMR (126 MHz, CDCl$_3$): δ 201.4, 200.6, 134.6, 134.5, 129.6, 129.3, 76.8, 70.5, 42.1, 30.5, 29.7.

3-(1-(4-bromophenyl)-2-nitroethyl)pentane-2,4-dione (**6c**)[1]

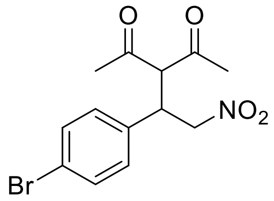

White crystalline solid, 96 % yield.

^{1}H NMR (500 MHz, CDCl$_3$): δ 7.50–7.42 (m, 2H), 7.13–7.03 (m, 2H), 4.66–4.57 (m, 2H), 4.33 (d, *J* = 10.7 Hz, 1H), 4.22 (ddd, *J* = 10.7, 7.3, 5.4 Hz, 1H), 2.30 (s, 3H), 1.98 (s, 3H).

^{13}C{^{1}H} NMR (126 MHz, CDCl$_3$): δ 201.4, 200.6, 135.1, 132.5, 129.6, 122.7, 77.9, 70.4, 42.2, 30.5, 29.7.

3-(1-(4-methoxyphenyl)-2-nitroethyl)pentane-2,4-dione (**6d**)[1]

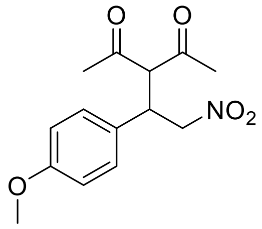

White crystalline solid, 96 % yield.

^{1}H NMR (500 MHz, CDCl$_3$): δ 7.16–7.05 (m, 2H), 6.89–6.79 (m, 2H), 4.65–4.53 (m, 2H), 4.33 (d, *J* = 10.9 Hz, 1H), 4.20 (ddd, *J* = 10.9, 7.0, 5.8 Hz, 1H), 3.77 (s, 3H), 2.29 (s, 3H), 1.94 (s, 3H).

^{13}C{^{1}H} NMR (126 MHz, CDCl$_3$): δ 201.9, 201.2, 159.5, 129.1, 127.6, 114.7, 78.5, 70.9, 55.2, 42.1, 30.4, 29.5.

3-(2-nitro-1-(*p*-tolyl)ethyl)pentane-2,4-dione (**6e**)[1]

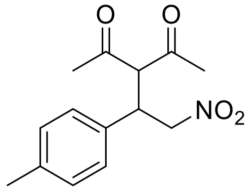

White crystalline solid, 98 % yield.

^{1}H NMR (500 MHz, CDCl$_3$): δ 7.15–7.10 (m, 2H), 7.09–7.04 (m, 2H), 4.65–4.56 (m, 2H), 4.35 (d, *J* = 10.9 Hz, 1H), 4.20 (ddd, *J* = 10.9, 7.7, 5.0 Hz, 1H), 2.30 (s, 3H), 2.29 (s, 3H), 1.94 (s, 3H).

$^{13}C\{^1H\}$ NMR (126 MHz, $CDCl_3$): δ 201.9, 201.1, 138.4, 132.8, 130.0, 127.8, 78.4, 70.8, 42.5, 30.5, 29.5, 21.1.

3-(1-(4-cyanophenyl)-2-nitroethyl)pentane-2,4-dione (**6f**)[3]

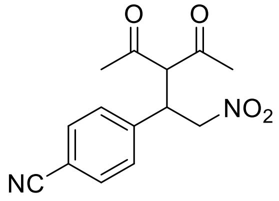

White crystalline solid, 95 % yield.

^1H NMR (500 MHz, $CDCl_3$): δ 7.67–7.63 (m, 2H), 7.37–7.32 (m, 2H), 4.73–4.61 (m, 2H), 4.37 (d, J = 10.5 Hz, 1H), 4.32 (ddd, J = 10.5, 7.7, 4.4 Hz, 1H), 2.32 (s, 3H), 2.02 (s, 3H).

$^{13}C\{^1H\}$ NMR (126 MHz, $CDCl_3$): δ 200.9, 200.0, 141.6, 133.0, 128.9, 118.0, 112.7, 77.4, 70.0, 42.6, 30.6, 29.9.

3-(1-(2-fluorophenyl)-2-nitroethyl)pentane-2,4-dione (**6g**)[2]

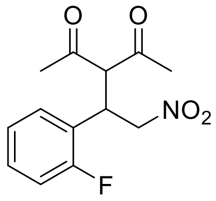

White crystalline solid, 98 % yield.

^1H NMR (500 MHz, $CDCl_3$): δ 7.36–7.23 (m, 1H), 7.20–7.15 (m, 1H), 7.13–7.05 (m, 2H), 4.77–4.69 (m, 1H), 4.67–4.61 (m, 1H), 4.53–4.44 (m, 2H), 2.29 (s, 3H), 2.02 (s, 3H).

$^{13}C\{^1H\}$ NMR (126 MHz, $CDCl_3$): δ 201.4, 200.8, 160.7 (d), 130.50 (d), 130.45 (d), 125 (d), 122.9 (d), 116.3, 76.6 (d), 69.0 (d), 37.9, 30.5, 29.3.

^{19}F NMR (471 MHz, $CDCl_3$): δ −116.1.

3-(1-(2-chlorophenyl)-2-nitroethyl)pentane-2,4-dione (**6h**)[4]

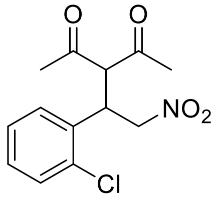

White crystalline solid, 99 % yield

^{1}H NMR (500 MHz, CDCl3): 7.45−7.40 (m, 1H), 7.26−7.21 (m, 2H), 7.19−7.15 (m, 1H), 4.87−4.58 (m, 4H), 2.28 (s, 3H), 2.04 (s, 3H) ppm.

^{13}C{^{1}H} NMR (126 MHz, CDCl$_3$): δ 201.9, 200.9, 133.8, 133.5, 130.7, 129.7, 129.0, 127.7, 77.5, 68.9, 38.9, 30.9, 28.6.

3-(1-(2-bromophenyl)-2-nitroethyl)pentane-2,4-dione (**6i**)[2]

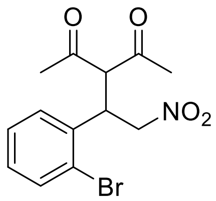

White crystalline solid, 96 % yield.

^{1}H NMR (500 MHz, CDCl$_3$): δ 7.63 (dd, J = 7.6, 1.4 Hz, 1H), 7.29 (td, J = 7.6, 1.4 Hz, 1H), 7.21–7.11 (m, 2H), 4.84 (dd, J = 12.3, 6.4 Hz, 1H), 4.74 (ddd, J = 10.2, 6.4, 4.0 Hz, 1H), 4.67 (dd, J = 12.3, 4.0 Hz, 1H), 4.61 (d, J = 9.8 Hz, 1H), 2.29 (s, 3H), 2.05 (s, 3H).

^{13}C{^{1}H} NMR (126 MHz, CDCl$_3$): δ 202.0, 200.9, 135.0, 134.0, 130.0, 128.3, 76.3, 69.1, 41.1, 31.0, 28.4.

3-(1-(2-methoxyphenyl)-2-nitroethyl)pentane-2,4-dione (**6j**)[1]

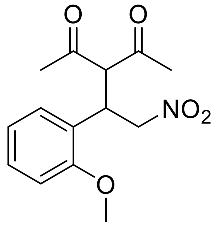

White crystalline solid, 97 % yield.

^{1}H NMR (500 MHz, CDCl$_3$): δ 7.29–7.23 (m, 1H), 7.08 (dd, J = 7.8, 1.7 Hz, 1H), 6.92–6.86 (m, 2H), 4.78 (dd, J = 12.2, 7.9 Hz, 1H), 4.65–4.55 (m, 2H), 4.52–4.46 (m, 1H), 3.89 (s, 3H), 2.28 (s, 3H), 1.94 (s, 3H).

^{13}C{^{1}H} NMR (126 MHz, CDCl$_3$): δ 202.3, 201.6, 157.0, 130.2, 129.8, 123.5, 121.2, 111.3, 76.6, 69.1, 55.5, 38.9, 30.5, 28.7.

3-(2-nitro-1-(2-nitrophenyl)ethyl)pentane-2,4-dione (**6k**)[4]

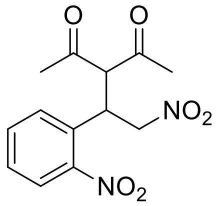

White crystalline solid, 98 % yield.

^{1}H NMR (500 MHz, CDCl$_3$): δ 7.94 (dd, *J* = 8.1, 1.4 Hz, 1H), 7.62–7.56 (m, 1H), 7.52–7.46 (m, 1H), 7.36 (dd, *J* = 7.9, 1.4 Hz, 1H), 4.98 (dd, *J* = 13.3, 7.1 Hz, 1H), 4.84 (dd, *J* = 13.3, 3.7 Hz, 1H), 4.74 (ddd, *J* = 8.8, 7.1, 3.7 Hz, 1H), 4.68 (d, *J* = 8.8 Hz, 1H), 2.32 (s, 3H), 2.14 (s, 3H).

^{13}C{^{1}H} NMR (126 MHz, CDCl$_3$): δ 201.6, 200.6, 149.8, 133.5, 131.2, 129.4, 129.3, 125.6, 76.6, 69.1, 37.2, 31.3, 29.3.

3-(1-(3-chlorophenyl)-2-nitroethyl)pentane-2,4-dione (**6l**)[4]

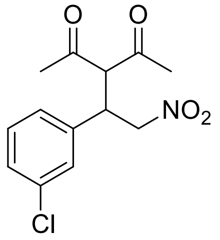

White crystalline solid, 99 % yield.

^{1}H NMR (500 MHz, CDCl$_3$): δ 7.31–7.26 (m, 2H), 7.23–7.18 (m, 1H), 7.12–7.06 (m, 1H), 4.69–4.58 (m, 2H), 4.35 (d, *J* = 10.6 Hz, 1H), 4.23 (ddd, *J* = 10.6, 7.7, 5.0 Hz, 1H), 2.30 (s, 3H), 2.00 (s, 3H).

^{13}C{^{1}H} NMR (126 MHz, CDCl$_3$): δ 201.4, 200.5, 138.2, 135.2, 130.6, 128.9, 128.2, 126.2, 77.7, 70.3, 42.4, 30.5, 29.8.

3-(1-(3-bromophenyl)-2-nitroethyl)pentane-2,4-dione (**6m**)[1]

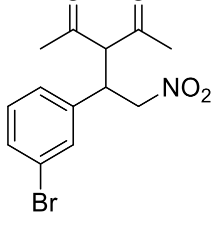

White crystalline solid, 97 % yield.

^{1}H NMR (500 MHz, $CDCl_3$): δ 7.48–7.39 (m, 1H), 7.40–7.33 (m, 1H), 7.25–7.17 (m, 1H), 7.16–7.10 (m, 1H), 4.72–4.56 (m, 2H), 4.35 (d, *J* = 10.6 Hz, 1H), 4.22 (ddd, *J* = 10.6, 7.8, 4.9 Hz, 1H), 2.30 (s, 3H), 2.01 (s, 3H).

$^{13}C\{^1H\}$ NMR (126 MHz, $CDCl_3$): δ 201.4, 200.5, 138.5, 131.8, 131.1, 130.9, 126.6, 123.3, 70.3, 42.3, 30.6, 29.8.

3-(1-(3-methoxyphenyl)-2-nitroethyl)pentane-2,4-dione (**6n**)[2]

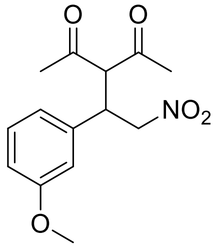

White crystalline solid, 87 % yield.

^{1}H NMR (500 MHz, $CDCl_3$): δ 7.30–7.21 (m, 1H), 6.84–6.80 (m, 1H), 6.77–6.74 (m, 1H), 6.73–6.69 (m, 1H), 4.70–4.53 (m, 2H), 4.37 (d, *J* = 10.8 Hz, 1H), 4.21 (ddd, *J* = 10.8, 7.8, 4.9 Hz, 1H), 3.78 (s, 3H), 2.30 (s, 3H), 1.97 (s, 3H).

$^{13}C\{^1H\}$ NMR (126 MHz, $CDCl_3$): δ 201.8, 201.0, 160.1, 137.6, 130.4, 119.9, 114.1, 113.6, 78.2, 70.6, 55.3, 42.8, 30.5, 29.6.

3-(1-(2,6-dichlorophenyl)-2-nitroethyl)pentane-2,4-dione (**6o**)[5]

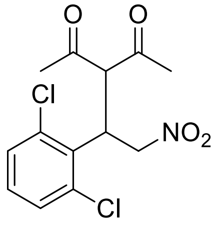

White crystalline solid, 94 % yield.

^{1}H NMR (500 MHz, $CDCl_3$): δ 7.42–7.35 (m, 1H), 7.32–7.28 (m, 1H), 7.21–7.15 (m, 1H), 5.43 (ddd, *J* = 11.2, 9.3, 5.0 Hz, 1H), 5.00 (dd, *J* = 12.6, 9.3 Hz, 1H), 4.88 (d, *J* = 11.2 Hz, 1H), 4.65 (dd, *J* = 12.6, 5.0 Hz, 1H), 2.30 (s, 3H), 2.03 (s, 3H).

$^{13}C\{^1H\}$ NMR (126 MHz, $CDCl_3$): δ 200.7, 200.4, 137.9, 134.6, 131.6, 130.2, 130.2, 129.7, 75.2, 68.3, 38.3, 29.6, 29.4.

3-(1-(2,4-dichlorophenyl)-2-nitroethyl)pentane-2,4-dione (**6p**)[1]

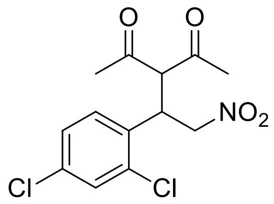

White crystalline solid, 95 % yield.

^{1}H NMR (500 MHz, CDCl$_3$): δ 7.39 (d, *J* = 2.2 Hz, 1H), 7.16 (dd, *J* = 8.4, 2.2 Hz, 1H), 7.04 (d, *J* = 8.4 Hz, 1H), 4.75 (dd, *J* = 12.4, 6.7 Hz, 1H), 4.62 (ddd, *J* = 10.0, 6.7, 4.0 Hz, 1H), 4.57 (dd, *J* = 12.4, 4.0 Hz, 1H), 4.48 (d, *J* = 9.7 Hz, 1H), 2.22 (s, 3H), 1.99 (s, 3H).

^{13}C{^{1}H} NMR (126 MHz, CDCl$_3$): δ 201.6, 200.5, 135.1, 134.5, 132.1, 130.5, 129.9, 128.0, 76.0, 68.8, 38.5, 30.9, 28.7.

3-(1-(2,5-dimethoxyphenyl)-2-nitroethyl)pentane-2,4-dione (**6q**)[5]

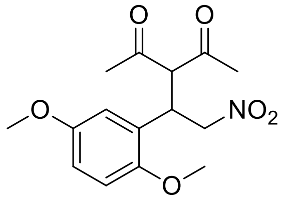

White crystalline solid, 96 % yield.

^{1}H NMR (300 MHz, CDCl$_3$): δ 6.87–6.73 (m, 2H), 6.68–6.61 (m, 1H), 4.78 (dd, *J* = 12.3, 8.0 Hz, 1H), 4.64–4.53 (m, 2H), 4.42 (ddd, *J* = 10.6, 8.0, 4.4 Hz, 1H), 3.84 (s, 3H), 3.72 (s, 3H), 2.27 (s, 3H), 1.96 (s, 3H).

^{13}C{^{1}H} NMR (126 MHz, CDCl$_3$): δ 202.2, 201.6, 153.6, 151.1, 124.5, 116.5, 114.0, 112.1, 76.5, 68.9, 55.9, 55.7, 39.3, 30.5, 29.0.

3-(2-nitro-1-(3,4,5-trimethoxyphenyl)ethyl)pentane-2,4-dione (**6r**)[6]

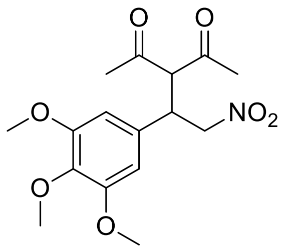

White crystalline solid, 92 % yield.

^{1}H NMR (500 MHz, CDCl$_3$): δ 6.37 (s, 2H), 4.70–4.55 (m, 2H), 4.37 (d, *J* = 10.6 Hz, 1H), 4.18 (ddd, *J* = 10.6, 7.8, 4.9 Hz, 1H), 3.83 (s, 6H), 3.81 (s, 3H), 2.30 (s, 3H), 1.99 (s, 3H).

$^{13}C\{^1H\}$ **NMR (126 MHz, CDCl3)**: δ 201.7, 201.0, 153.7, 138.0, 131.6, 105.1, 78.2, 70.7, 60.8, 56.2, 43.0, 30.5, 29.6.

3-(1-(furan-2-yl)-2-nitroethyl)pentane-2,4-dione (**6s**)[1]

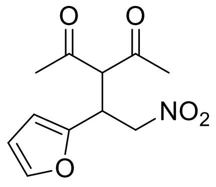

Colorless oil, 98 % yield.

^1H **NMR (500 MHz, CDCl3)**: δ 7.36 (d, J = 1.9 Hz, 1H), 6.30 (dd, J = 3.3, 1.9 Hz, 1H), 6.18 (d, J = 3.3 Hz, 1H), 4.67 (d, J = 5.7 Hz, 2H), 4.45–4.30 (m, 2H), 2.28 (s, 3H), 2.09 (s, 3H).

$^{13}C\{^1H\}$ **NMR (126 MHz, CDCl3)**: δ 201.52, 200.87, 149.45, 142.92, 110.83, 108.86, 75.83, 67.83, 36.57, 30.67, 29.37.

3-(1-nitropentan-2-yl)pentane-2,4-dione (**6t**)[5]

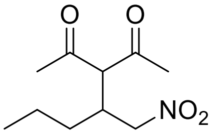

Colorless oil, 85 % yield.

^1H **NMR (500 MHz, CDCl3)**: δ 4.53–4.44 (m, 2H), 3.99 (d, J = 8.6 Hz, 1H), 2.91–2.77 (m, 1H), 2.27 (s, 3H), 2.25 (s, 3H), 1.51–1.18 (m, 4H), 0.90 (t, J = 7.1 Hz, 3H).

$^{13}C\{^1H\}$ **NMR (126 MHz, CDCl3)**: δ 202.8, 202.4, 75.8, 69.3, 36.8, 31.6, 31.0, 29.9, 19.9, 13.7.

methyl 4-acetyl-5-oxohexanoate[7]

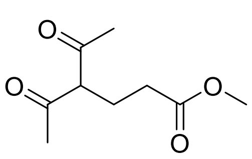

Colorless oil, 96 % yield.

^1H **NMR (500 MHz, CDCl3)**: δ 3.68 (t, J = 7.1 Hz, 1H), 3.61 (s, 3H), 2.52 (t, J = 7.1 Hz, 2H), 2.14 (s, 6H), 2.07 (m, 2H).

$^{13}C\{^1H\}$ **NMR (126 MHz, CDCl3)**: δ 207.0, 206.2, 173.1, 68.3, 51.9, 28.1, 25.1, 18.6.

2-(2-nitro-1-phenylethyl)-1-phenylbutane-1,3-dione[8]

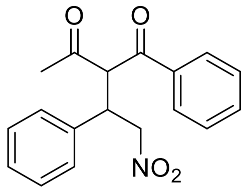

Yellow solid as a mixture of diastereoisomers, 95 % yield.

^{1}H NMR (500 MHz, $CDCl_3$): δ 8.01 (d, *J* = 7.3 Hz, 1H), 7.81 (d, *J* = 7.7 Hz, 1H), 7.12–7.65 (m, 8H), 5.18 (2 x d, *J* = 9.9, 9.8 Hz, 1H), 4.63–4.89 (m, 2H), 4.39–4.57 (m, 1H), 2.22 (s, 2H), 1.93 (s, 1H).

^{13}C{^{1}H} NMR (126 MHz, $CDCl_3$): δ 201.8, 200.9, 194.2, 193.9, 136.7, 136.4, 136.3, 136.2, 134.6, 134.2, 129.4, 129.2, 129.1, 129.1, 128.7, 128.3, 128.3, 128.2, 78.3, 78.2, 65.5, 64.9, 43.6, 43.4, 29.9, 28.6.

ethyl 2-acetyl-4-nitro-3-phenylbutanoate[8]

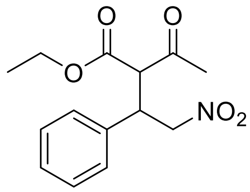

White solid as a mixture of diastereoisomers, 97 % yield.

^{1}H NMR (500 MHz, $CDCl_3$): δ 7.19–7.34 (m, 5H), 4.74–4.84 (m, 2H), 3.93–4.26 (m, 4H), 2.05–2.29 (2 x s, 3H), 0.97–1.30 (2 x t, *J* = 7.1 Hz, 3H).

^{13}C{^{1}H} NMR (126 MHz, $CDCl_3$): δ 201.3, 200.4, 167.7, 167.0, 136.6, 136.6, 129.3, 129.1, 128.5, 128.4, 128.1, 128.0, 78.0, 77.9, 62.3, 62.1, 62.1, 61.8, 42.7, 42.5, 30.4, 30.2, 14.1, 13.8.

(1,3-dinitropropan-2-yl)benzene[9]

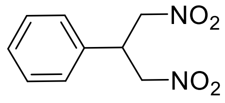

Brown oil, 98 % yield.

^{1}H NMR (500 MHz, $CDCl_3$): δ 7.36–7.29 (m, 3H), 7.2–7.17 (m, 2H), 4.76–4.65 (m, 4H), 4.3–4.22 (quin, *J* = 7.21 Hz, 1H).

^{13}C{^{1}H} NMR (126 MHz, $CDCl_3$): δ 134.5, 129.7, 129.2, 127.6, 76.9, 41.9.

CRYSTALLOGRAPHIC PART

Selected crystals attached to MiTeGen MicroLoops with aid of silicone grease (Bayer) were measured on an Agilent New Gemini Dual four-circle kappa-goniometer diffractometer employing a graphite-monochromatized fine-focus sealed Cu *K*α X-ray source and an Atlas CCD area detector. During the measurement crystals were kept at 150 K by a cold nitrogen stream of an Oxford Cryosystems Cryostream 800 cooling device. *CrysAlis^{Pro}* software[10] was used for data collection and processing, which included an empirical absorption correction using spherical harmonics, implemented in *SCALE3 ABSPACK*, and an analytical numeric absorption correction using a multifaceted crystal model.[11] *Olex2* (v. 1.5)[12] was used for crystal structure solution and refinement (full-matrix least-squares minimization) employing *SHELXT*[13] and *SHELXL*[14] (v. 2019/2), respectively. Molecular graphics were created with *Diamond*[15] program.

Hydrogen atoms in the crystal structure of **3**·0.5acac (Figure S1) were positioned on residual density peaks found after all the heavier atoms were included in the model. Their position and isotropic thermal parameters were refined freely. The exception are the methyl group hydrogen atoms on atoms C29 and C30A, C30B of the disordered cocrystallized acac, which were included at calculated positions and refined using a riding model. The distance to hydrogen atom H32A on C32A was restrained (DFIX) to value 0.95 Å.

Hydrogen atoms in the crystal structure of **3**·0.5C_6H_{14} (Figure S3) were included at calculated positions and refined using a riding model. The hydrogen atom on C27 of the acetylacetonate anion ligand was refined freely with isotropic thermal parameter to verify the deprotonation of acetylacetone molecule. A somewhat elongated thermal ellipsoid of the atom C16 in the crystal structure of **3**·0.5C_6H_{14} might indicate a possible disorder. However, a disordered model where this atom was split did not significantly improve the *R*-values and displayed distorted geometrical parameters. A non-disordered structure model is therefore reported.

Table S1. Summary of crystal data and structure refinements

**Compound**	**3**·0.5acac	**3**·0.5C_6H_{14}
Formula	$C_{31.5}H_{54}O_3P_2Pd$	$C_{32}H_{57}O_2P_2Pd$
F_w	649.08	642.11
T [K]	150.0(1)	150.0(1)
Crystal system	Monoclinic	Triclinic
Space group	P−1	P−1
a [Å]	8.6620(3)	11.2071(4)
b [Å]	12.8231(5)	11.6280(4)
c [Å]	16.0688(6)	14.5001(5)
α [°]	79.513(3)	73.720(3)
β [°]	75.257(4)	81.114(3)
γ [°]	74.019(3)	66.590(3)
V [Å^3]	1647.28(11)	1662.53(11)
Z	2	2
ρ_{calc} [g/cm^3]	1.309	1.283
Crystal size [mm]	0.33 × 0.21 × 0.14	0.36 × 0.19 × 0.07
Radiation type	Cu $K\alpha$	Cu $K\alpha$
λ [Å]	1.54184	1.54184
μ [mm^{-1}]	5.675	5.592
F(000)	686	682
θ_{max} [°]	72.686	76.549
Index ranges	$-10 \le h \le 10$	$-14 \le h \le 14$
	$-15 \le k \le 15$	$-14 \le k \le 14$
	$-19 \le l \le 19$	$-18 \le l \le 18$
Reflections collected	36163	35250
Independent reflections	6422	6805
Reflections with [$I > 2\sigma(I)$]	6257	6257
R_{int}	0.0262	0.0398
R_{sigma}	0.0138	0.0243
Data/restraints/parameters	6422/0/580	6805/0/353
S	1.045	1.054
R_1, wR_2 [$I > 2\sigma(I)$]	0.0223, 0.0572	0.0334, 0.0856
R_1, wR_2 [all data]	0.0230, 0.0578	0.0372, 0.0889
$\Delta\rho_{min}$, $\Delta\rho_{max}$ [eÅ^{-3}]	−0.416, 0.479	−0.455, 1.083
CCDC deposition number [a]	2432904	2432905

[a] CCDC 2432904 and 2432905 contain the supplementary crystallographic data for this paper. These data can be obtained free of charge from The Cambridge Crystallographic Data Centre (CCDC) via www.ccdc.cam.ac.uk/structures.

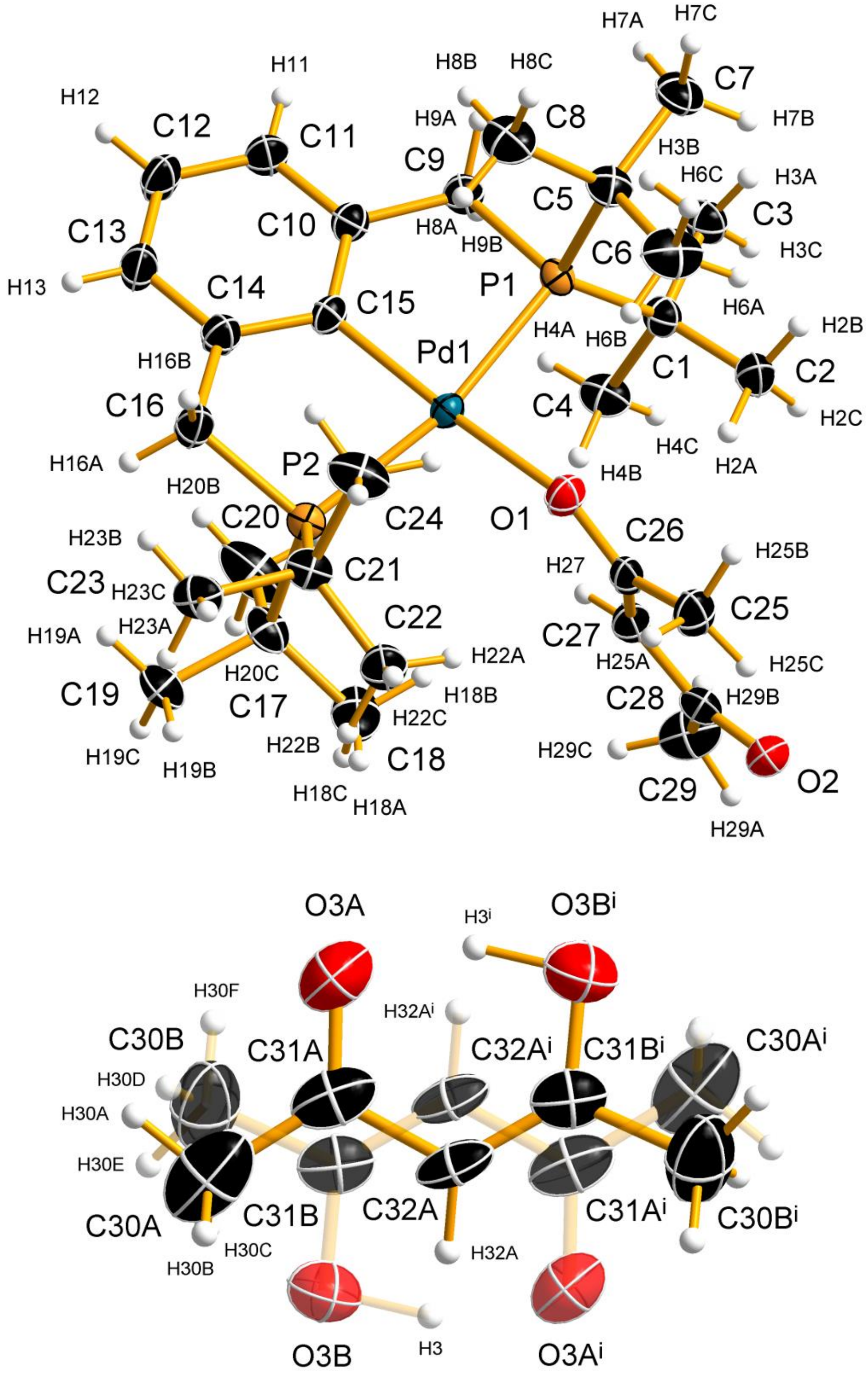

Figure S1. The atom numbering scheme and the asymmetric unit of the crystal structure of **3**·0.5acac. Thermal ellipsoids are plotted at the 50% probability level and hydrogen atoms are depicted as small spheres of arbitrary radius. The asymmetric unit also contains one half of cocrystallised acetylacetone molecule, which is disordered over two sites and sits on an inversion centre. Symmetry code: (i) $-x$, $-y$, $-z$.

Table S2. Selected bond distances, angles, and intermolecular hydrogen-bond geometry (Å, °) in **3**·0.5acac

**Bond Lengths** [Å]							
				t-Bu		acac	
Pd1−P1	2.3172(4)	C9−C10	1.505(2)	C1−C2	1.530(3)	O1−C26	1.286(2)
Pd1−P2	2.3241(4)	C10−C11	1.394(2)	C1−C3	1.536(2)	O2−C28	1.236(2)
Pd1−O1	2.1050(12)	C11−C12	1.386(3)	C1−C4	1.536(3)	C25−C26	1.507(2)
Pd1−C15	2.0105(16)	C12−C13	1.381(3)	C5−C6	1.535(3)	C26−C27	1.374(3)
		C13−C14	1.391(3)	C5−C7	1.530(3)	C27−C28	1.430(3)
P1−C1	1.8727(17)	C14−C15	1.415(2)	C5−C8	1.537(3)	C28−C29	1.519(3)
P1−C5	1.8715(18)	C14−C16	1.509(2)	C17−C18	1.526(3)		
P1−C9	1.8400(18)	C15−C10	1.411(2)	C17−C19	1.528(3)	acac	
P2−C16	1.8315(18)			C17−C20	1.535(3)	O3A−C31A	1.249(8)
P2−C17	1.8747(18)			C21−C22	1.535(3)	O3B−C31B	1.314(7)
P2−C21	1.8716(17)			C21−C23	1.531(3)	C30A−C31A	1.513(13)
				C21−C24	1.541(3)	C31A−C32A	1.419(9)
						C30B−C31B	1.478(10)
						C32A−C31B^{i}	1.348(9)

**Bond Angles** [°]					
P1−Pd1−P2	167.210(16)	Pd1−P1−C9	103.00(6)	Pd1−P2−C16	100.52(6)
C15−Pd1−P1	83.17(5)	Pd1−P1−C1	118.04(6)	Pd1−P2−C17	116.02(6)
C15−Pd1−P2	84.06(5)	Pd1−P1−C5	111.77(6)	Pd1−P2−C21	116.00(6)
O1−Pd1−P1	97.74(4)	C1−P1−C5	113.40(8)	C17−P2−C21	112.96(8)
O1−Pd1−P2	94.94(4)				
O1−Pd1−C15	168.66(6)	P1−C9−C10	109.15(12)	P2−C16−C14	109.59(12)
Pd1−C15−C10	122.34(12)	C9−P1−C1	103.33(8)	C16−P2−C17	104.81(9)
Pd1−C15−C14	120.34(12)	C9−P1−C5	105.46(9)	C16−P2−C21	104.14(9)
Pd1−O1−C26	133.65(12)			C25−C26−C27	123.16(17)
O1−C26−C25	113.44(17)	O2−C28−C29	117.80(18)	C26−C27−C28	128.87(17)
O1−C26−C27	123.38(16)	O2−C28−C27	126.72(19)	C27−C28−C29	115.54(18)

D*−H···*A	***D*−H**	**H···*A***	***D*···*A***	***D*−H···*A***
C25−H25C···O2	0.97(3)	2.24(3)	2.982(3)	132(2)
O3B−H3···O3A^{i}	1.12(7)	1.56(7)	2.510(6)	139(6)

Symmetry code: (i) −*x*, −*y*, −*z*.

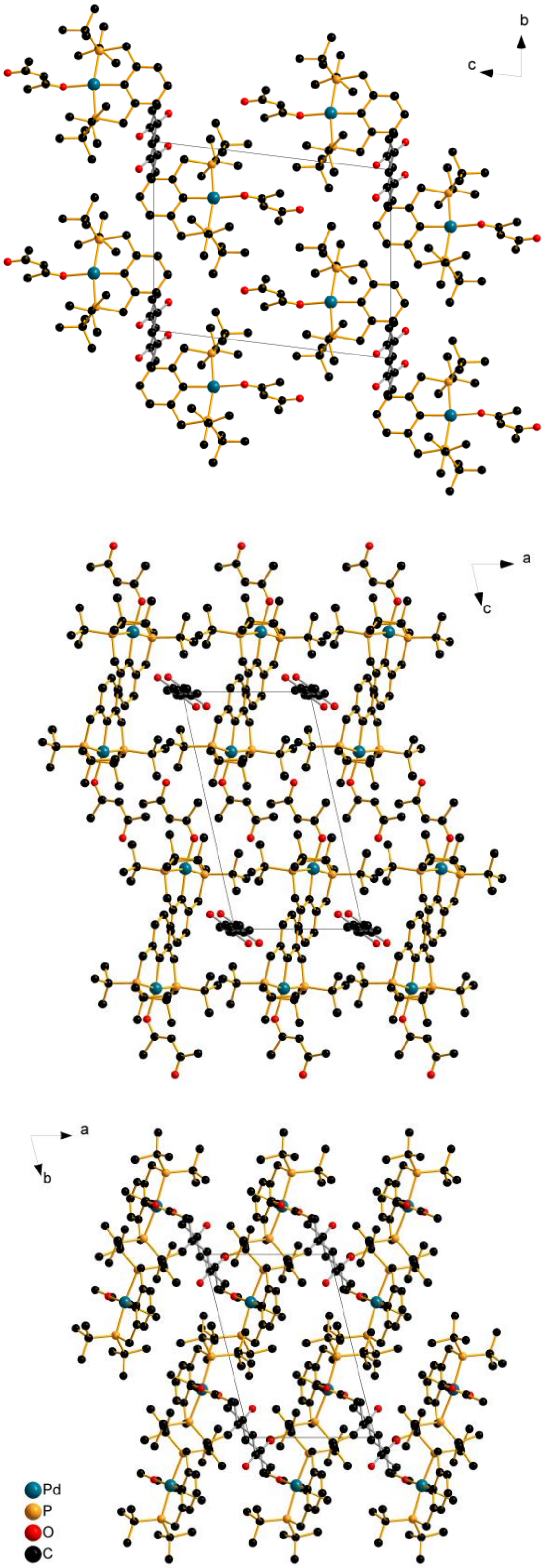

Figure S2. The crystal packing and the unit cell of **3**·0.5acac crystal structure viewed along the *a*-crystallographic axis (top), *b*-crystallographic axis (middle), and *c*-crystallographic axis (bottom). Hydrogen atoms have been omitted for clarity.

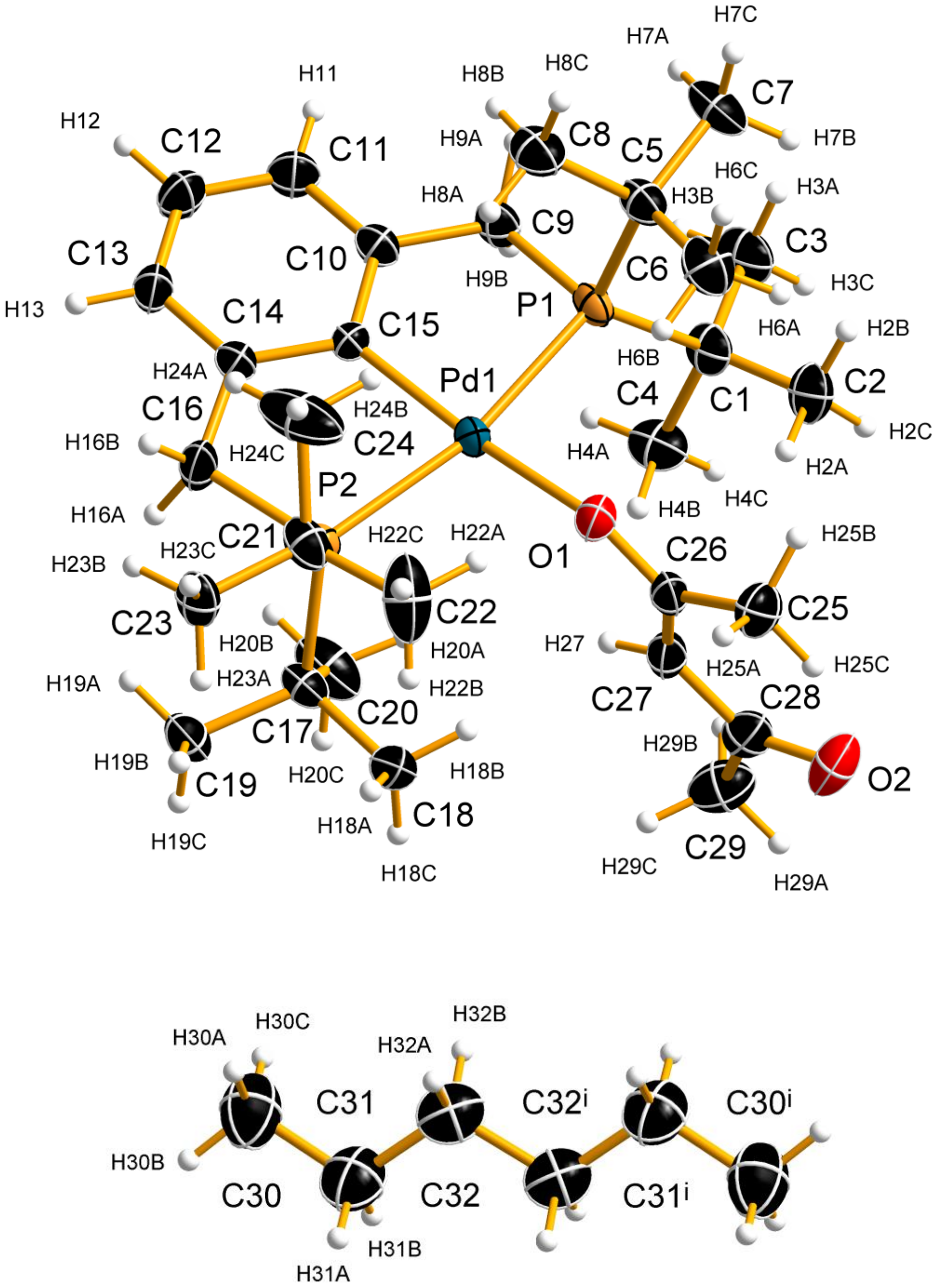

Figure S3. The atom numbering scheme and the asymmetric unit of the crystal structure of complex **3**·0.5C_6H_{14}. Thermal ellipsoids are plotted at the 50% probability level and hydrogen atoms are depicted as small spheres of arbitrary radius. The asymmetric unit also contains one half of hexane solvent molecule, which sits on an inversion center.

Symmetry code: (i) −*x*, −*y*+1, −*z*.

Table S3. Selected bond distances, angles, and intermolecular hydrogen-bond geometry (Å, °) in **3**·0.5C_6H_{14}

**Bond Lengths** [Å]							
				t-Bu		acac	
Pd1−P1	2.3256(6)	C9−C10	1.506(4)	C1−C2	1.525(4)	O1−C26	1.281(3)
Pd1−P2	2.3202(6)	C10−C11	1.389(4)	C1−C3	1.528(4)	O2−C28	1.237(4)
Pd1−O1	2.1089(18)	C11−C12	1.382(4)	C1−C4	1.540(4)	C25−C26	1.507(4)
Pd1−C15	2.015(2)	C12−C13	1.381(4)	C5−C6	1.542(4)	C26−C27	1.379(4)
		C13−C14	1.392(4)	C5−C7	1.527(4)	C27−C28	1.424(4)
P1−C1	1.883(3)	C14−C15	1.407(3)	C5−C8	1.533(4)	C28−C29	1.512(4)
P1−C5	1.869(3)	C14−C16	1.504(4)	C17−C18	1.534(4)		
P1−C9	1.835(3)	C15−C10	1.416(3)	C17−C19	1.535(4)	hex	
P2−C16	1.836(3)			C17−C20	1.530(4)	C30−C31	1.493(6)
P2−C17	1.876(3)			C21−C22	1.508(4)	C31−C32	1.490(5)
P2−C21	1.870(3)			C21−C23	1.524(4)	C32−C32^{i}	1.515(7)
				C21−C24	1.551(4)		

**Bond Angles** [°]					
P1−Pd1−P2	167.71(2)	Pd1−P1−C9	102.11(9)	Pd1−P2−C16	103.01(9)
C15−Pd1−P1	83.88(7)	Pd1−P1−C1	121.08(9)	Pd1−P2−C17	115.45(9)
C15−Pd1−P2	83.89(7)	Pd1−P1−C5	111.04(9)	Pd1−P2−C21	114.05(9)
O1−Pd1−P1	95.81(6)	C1−P1−C5	112.11(12)	C17−P2−C21	113.74(12)
O1−Pd1−P2	96.02(5)				
O1−Pd1−C15	170.01(8)	P1−C9−C10	109.91(18)	P2−C16−C14	110.28(19)
Pd1−C15−C10	121.55(18)	C9−P1−C1	103.25(13)	C16−P2−C17	103.11(14)
Pd1−C15−C14	121.64(18)	C9−P1−C5	105.11(13)	C16−P2−C21	105.65(15)
Pd1−O1−C26	131.89(18)			C25−C26−C27	122.7(2)
O1−C26−C25	114.1(3)	O2−C28−C29	117.7(3)	C26−C27−C28	128.5(3)
O1−C26−C27	123.2(2)	O2−C28−C27	126.5(3)	C27−C28−C29	115.8(3)

D*−H···*A	***D*−H**	**H···*A***	***D*···*A***	***D*−H···*A***
C25−H25C···O2	0.98	2.18	2.953(4)	134.7

Symmetry code: (i) −*x*, −*y*+1, −*z*.

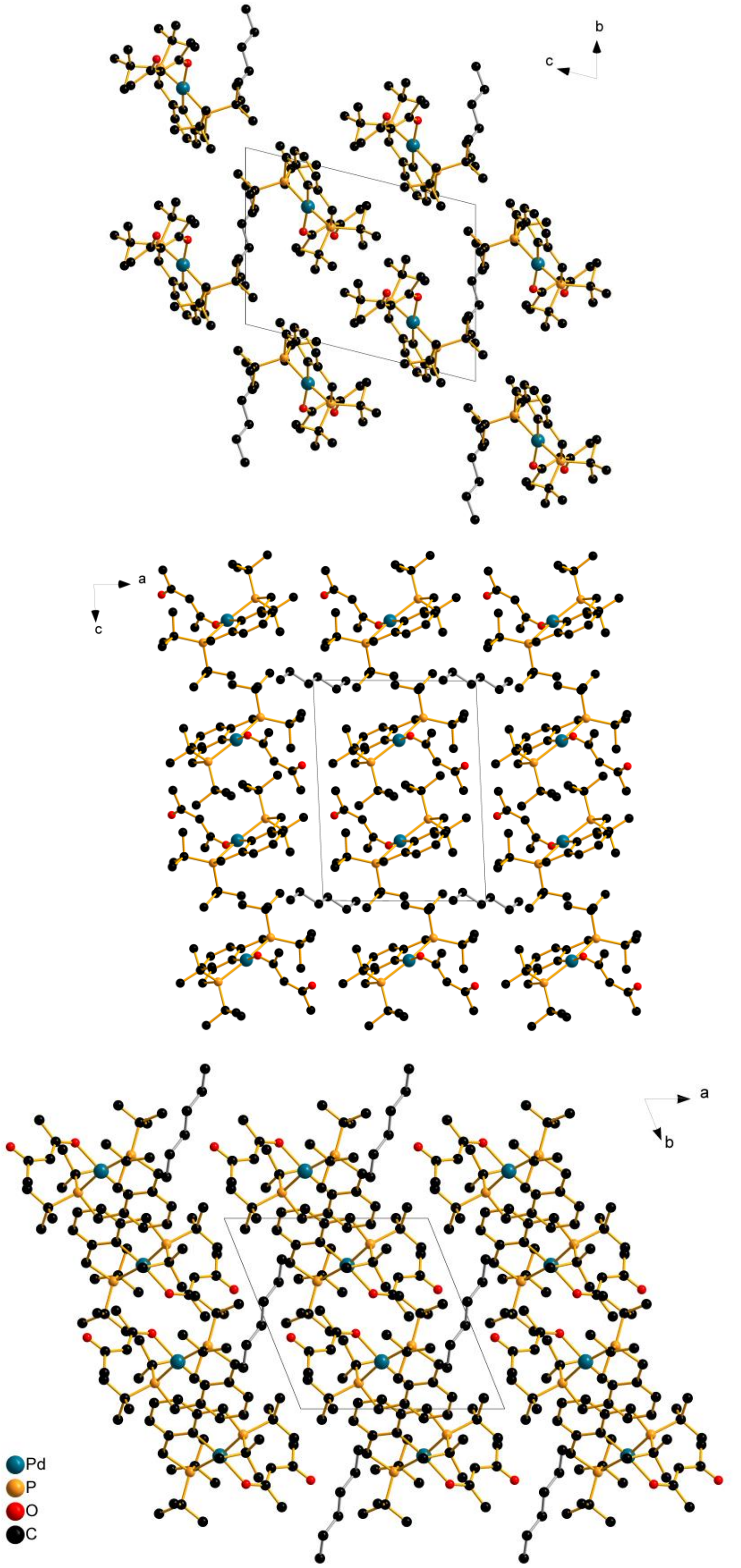

Figure S4. The crystal packing and the unit cell of **3**·$0.5C_6H_{14}$ crystal structure viewed along the *a*-crystallographic axis (top), *b*-crystallographic axis (middle), and *c*-crystallographic axis (bottom). Hydrogen atoms have been omitted for clarity.

SUPPORTING REFERENCES

(1) Kasaplar, P.; Riente, P.; Hartmann, C.; Pericàs, M. A. A Polystyrene-Supported, Highly Recyclable Squaramide Organocatalyst for the Enantioselective Michael Addition of 1,3-Dicarbonyl Compounds to β-Nitrostyrenes. *Adv. Synth. Catal.* **2012**, *354*, 2905–2910.

(2) Vural, U.; Durmaz, M.; Sirit, A. A novel calix[4]arene-based bifunctional squaramide organocatalyst for enantioselective Michael addition of acetylacetone to nitroolefins. *Org. Chem. Front.* **2016**, *3*, 730–736.

(3) Zenz, I.; Mayr, H. Electrophilicities of *trans*-β-Nitrostyrenes. *J. Org. Chem.* **2011**, *76*, 9370–9378.

(4) Wang, Y.-F.; Chen, R.-X.; Wang, K.; Zhang, B.-B.; Li, Z.-B.; Xu, D.-Q. Fast, solvent-free and hydrogen-bonding-mediated asymmetric Michael addition in a ball mill. *Green Chem.* **2012**, *14*, 893–895.

(5) Ričko, S.; Svete, J.; Štefane, B.; Perdih, A.; Golobič, A.; Meden, A.; Grošelj, U. 1,3-Diamine-Derived Bifunctional Organocatalyst Prepared from Camphor. *Adv. Synth. Catal.* **2016**, *358*, 3786–3796.

(6) Chahal, M. K.; Payne, D. T.; Matsushita, Y.; Labuta, J.; Ariga, K.; Hill, J. P. Molecular Engineering of β-Substituted Oxoporphyrinogens for Hydrogen-Bond Donor Catalysis. *Eur. J. Org. Chem.* **2020**, *2020*, 82–90.

(7) Alexandrova, L. A.; Jasko, M. V.; Belobritskaya, E. E.; Chudinov, A. V.; Mityaeva, O. N.; Nasedkina, T. V.; Zasedatelev, A. S.; Kukhanova, M. K. New Triphosphate Conjugates Bearing Reporter Groups: Labeling of DNA Fragments for Microarray Analysis. *Bioconjugate Chem.* **2007**, *18*, 886−893.

(8) Chahal, M. K.; Payne, D. T.; Matsushita, Y.; Labuta, J.; Ariga, K.; Hill, J. P. Molecular Engineering of β-Substituted Oxoporphyrinogens for Hydrogen-Bond Donor Catalysis. *Eur. J. Org. Chem.* **2020**, *2020*, 82–90.

(9) Poe, S. L.; Kobašlija, M.; McQuade, D. T. Microcapsule Enabled Multicatalyst System. *J. Am. Chem. Soc.* **2006**, *128*, 15586−15587.

(10) Rigaku OD, *CrysAlis PRO*, Rigaku Corporation, Wrocław, Poland, **2021**.

(11) R. C. Clark, J. S. Reid, *Acta Crystallogr.* **1995**, *A51*, 887–897.

(12) O. V. Dolomanov, L. J. Bourhis, R. J. Gildea, J. A. K. Howard, H. Puschmann, *J. Appl. Cryst.* **2009**, *42*, 339–341.

(13) G. M. Sheldrick, *Acta Crystallogr.* **2015**, *A71*, 3–8.

(14) G. M. Sheldrick, *Acta Crystallogr.* **2015**, *C71*, 3–8.

(15) K. Brandenburg, *Diamond* – Crystal and Molecular Structure Visualization, v. 3.2, Crystal Impact GbR, Bonn, Germany, **2018**.
